# A Novel Method for Identifying Parkin Binding Agents in Complex Preparations of Herbal Medicines

**DOI:** 10.1155/2022/3260243

**Published:** 2022-01-18

**Authors:** Feng-Jiao Li, Fan Zhang, Xu-Dong He, Xin Liu, Jian-Kang Mu, Min Yang, Yan-Qin Li, Wen Gu, Jie Yu, Xing-Xin Yang

**Affiliations:** ^1^College of Pharmaceutical Science, Yunnan University of Chinese Medicine, 1076 Yuhua Road, Kunming 650500, China; ^2^Zhaotong City Institute of Gastrodia Elata, Zhaotong 657000, China; ^3^Yunnan Key Laboratory of Southern Medicine Utilization, 1076 Yuhua Road, Kunming 650500, China; ^4^Beijing Entry-Exit Inspection and Quarantine Bureau, Beijing 100026, China

## Abstract

Parkin is a crucial E3 ubiquitin ligase for initiating mitophagy through the PINK1/Parkin pathway. Regulating the expression and activity of parkin can remedy mitophagy and human disease. We developed an efficient method to isolate natural parkin ligands from herbal medicines by combining centrifugal ultrafiltration and liquid chromatography/mass spectrometry. The heterologous expression technology identified functionally active and pure parkin proteins. After evaluating the reliability of the method using DL-selenomethionine and DL-dithiothreitol as positive controls, this method was successfully applied to capture parkin ligands from Polygoni Cuspidati Rhizoma et Radix and Sophorae Flavescentis Radix. LC/MS identified seven novel parkin-targeting compounds, namely, 7,4′-dihydroxy-5-methoxy-8-(*γ*, *γ*-dimethylallyl)-flavanone, kushenol I, kurarinone, sophoraflavanone G, torachrysone-8-O-glucoside, apigenin, and emodin, supported by the molecular docking analysis. Five of the seven novel compounds (kushenol I, kurarinone, sophoraflavanone G, apigenin, and emodin) can activate parkin in *in vitro* autoubiquitination assays. Meanwhile, kushenol I and kurarinone had antisteatosis activity in fat emulsion-damaged human hepatocytes. These results confirmed the effectiveness of the method for identifying parkin ligands from complex preparations, useful to advance drug discovery from medicinal herbs.

## 1. Introduction

Parkin is an E3 ubiquitin ligase that localizes to the cytoplasm and mitochondria and plays a key role in the degradation of cytotoxic proteins through the ubiquitin-proteasome system. The dysregulation of parkin is linked to an array of disease states, including Parkinson's disease, cancer, liver disease, heart disease, skeletal muscle dysfunction, and antimicrobial activity. Through mitophagy, parkin regulates mitochondrial morphology and function in response to swelling and cristae fracture. Consequently, parkin is a prominent pharmacological target for drug development. However, no effective parkin ligands have been identified with clinical efficacy to date.

Herbal medicines (HMs) contain important compounds for new drug discovery due to their structural diversity, low toxicity, and numerous sources. HMs, including *Cinnamomum cassia* Presl [1] and *Rhodiola rosea L* [2], regulate parkin expression and mitophagy. HMs comprise several biologically active constituents. The classic procedure for discovering target compounds from HMs involves extraction and fishing, followed by the pharmacological screening of the purified substances. This method is time-consuming, labor-intensive, expensive, and often inefficient for directly screening bioactive compounds from natural samples. More recently, high-throughput screening methods [3] identified parkin ligands, although these are often unsuitable for directly determining multiple ligands from complex mixtures. Further development of efficient strategies is required to identify specific parkin ligands from complex samples.

Many experimental techniques, including biochromatography, centrifugal ultrafiltration (CU), centrifugal sedimentation, dialysis, magnetic separation, and hollow fiber adsorption, have been applied to fish ligands bound to biomacromolecules [4]. Biochromatography has the characteristics of both chromatographic separation and biological activity but remains disadvantages, such as the complicated preparation procedure and not-amenable bioactivity maintenance of stationary phase. Centrifugal sedimentation has the advantages of concise operational process and low cost of analysis. However, some inactive ingredients are not easily removed by centrifugal sedimentation. Additionally, some bound active ingredients are easily dissociated during the impurity washing process. Dialysis can be combined online with analytical system to detect active substances, and concentration changes of active compounds can be monitored in real time, but it is unavailable for sample enrichment, resulting in low sensitivity. Magnetic separation has a simple and efficient operational process using a magnetic field, but the target protein may be denatured or its three-dimensional configuration may be changed when the target protein is coupled to magnetic beads. Hollow fiber adsorption is a rapid and inexpensive process. However, the target adsorbed on the inner wall of the hollow fiber has a short survival time, and only few targets are adsorbed, restricting the sensitivity of this method. CU is the preferred technique for fishing biomacromolecule-bound ligands [5–9] because the technique is simple to operate, fast, and highly dependable. Liquid chromatography/mass spectrometry (LC/MS) is widely employed to separate and identify target constituents in complex samples [5–9]. Combining LC/MS with CU permits the efficient identification of target constituents in HMs. However, no methods have been reported for the direct identification of parkin ligands from complex mixtures.

Some HMs regulate parkin expression and mitophagy, thus, treating diseases such as *Magnolia officinalis* Rehd.et Wils. [10], Acanthopanax senticosus [11], Chen Formula [12], Sophorae Flavescentis Radix [13] (SFR), and Polygoni Cuspidati Rhizoma et Radix [14] (PCRR). This study developed a rapid and efficient fishing method combining CU with LC/MS to identify parkin ligands from Polygoni Cuspidati Rhizoma et Radix (PCRR) and Sophorae Flavescentis Radix (SFR). In this method, fractions containing parkin ligands were isolated using CU and subjected to LC/MS analysis for separation and identification. Pharmacological verification showed that the method is effective and efficient for rapid fishing of parkin ligands from complex samples (Figure S1). The technique holds utility for an in-depth and comprehensive assessment of the mechanism of action of medicinal herbs as lead compounds.

## 2. Material and Methods

### 2.1. Chemicals, Reagents, and Materials

DL-selenomethionine (ST), amoxicillin (AC), DL-dithiothreitol (DTT), and fenofibrate (FB) were purchased from Shanghai Yuanye Biotechnology Co., Ltd. (Shanghai, China). Kushenol I (K2), kurarinone (K3), and sophoraflavanone G (K4) were purchased from Wuhan Chemstan Biotechnology Co., Ltd. (Wuhan, China). Torachrysone-8-O-glucoside (H1), apigenin (H2), and emodin (H4) were purchased from Chengdu Pufeide Biological Technology Co., Ltd. (Chengdu, China). The purity of all reference substances is greater than 98%. Park2 plasmid was purchased from Shanghai Hewu Biotechnology Co., Ltd. (Shanghai, China). Coomassie brilliant Blue Gmur250, T4DNA ligase, *E. coli* BL21 (DE3), and 30% acrylamide were purchased from Beijing Solarbio Technology Co. Ltd. (Beijing, China). *Hin*dIII, *Eco*RI, and Protein Marker 170 were purchased from Thermo Fisher Scientific (MA, USA). Isopropyl IPTG was purchased from BioFroxx (Berlin, Germany). Competent *E. coli* DH5*α* cells were purchased from TaKaRa (Kusatsu-Shiga, Japan). DNA Marker2000, 2× powerTapPCR MasterMix, and nucleic acid dyes were purchased from Beijing Baitaike Biotechnology Co., Ltd. (Beijing, China). The pCMV-HA-Parkin plasmid was purchased from Shanghai Hewu Biotechnology Co., Ltd. (Shanghai, China). Ammonium persulfate (APS) and Nemerol Nomenclature- (TEMED-) Tetramethylethylenediamine were purchased from Shenggong Bioengineering Co., Ltd. (Shanghai, China). Sodium dodecyl sulfate (SDS) was purchased from Aladdin Reagent Co., Ltd. (Shanghai, China). His-tag protein purification kits and BCA assays were purchased from Shanghai Biyuntian Biotechnology Co., Ltd. (Shanghai, China). FK2 antibodies and TCL chemiluminescence detection reagent were purchased from Millipore (MA, USA). HRP-labeled goat anti-mouse IgG secondary antibodies were purchased from Proteintech Company (IL, USA). Tris base was purchased from Angus Company (Hong Kong, China). Glycine was purchased from Amresco Company (CA, USA). Yunnan Provincial Hospital of Traditional Chinese Medicine (Kunming, China) provided the fat emulsion. Commercial kits for the determination of triglyceride (TG), total cholesterol (TC), alanine transaminase (ALT), aspartate transaminase (AST), and ATP synthase (ATPase) were purchased from Nanjing Jiancheng Bioengineering Institute (Nanjing, China). Sophorae Flavescentis Radix (SFR) and Polygoni Cuspidati Rhixoma et Radix (PCRR) were purchased from the Traditional Chinese Medicine dispensary of Yunnan University of Chinese Medicine (Kunming, China).

### 2.2. Preparation of Parkin Protein

#### 2.2.1. Plasmid Construction

Full-length pCMV-HA-Parkin cDNA (Genebank, [NM004562]) was designed using Primer premier 5.0 (Premier Biosoft, CA, USA). Upstream and downstream primer sequences were as follows: 5′-AGGGAATTCATGATAGTGTTTGTCAGGTTCAACT-3′ and 5′-GGCAAGCTTCTACACGTCGAACCAGTGGTCCCCC-3′. Parkin was cloned into pMD18-T and PCR amplified under the following conditions: 95°C for 5 min 30 s; 94°C for 1 min, 30 cycles at 58°C for 40 s, and 72°C for 2 min; and final extension at 72°C for 10 min.

#### 2.2.2. Protein Expression and Purification

*Park2* was cloned into pET-28a (Thermo Fisher Scientific, MA, USA) and transformed into *Escherichia coli* BL21 (DE3). Cells were grown in Luria broth supplemented with 500 mM zinc chloride at 37°C until the OD_600_ values reached 0.4. Expression was induced with 25 mM IPTG for 12 h at 16°C. Using the bacterial protein extraction kit (Jiangsu, China), the total protein was extracted from the collected bacteria, following the manufacturer's protocol. Pure protein was flash-frozen in liquid nitrogen and stored at -80°C. Coomassie blue staining assessed the purity of the parkin sample [15]. Parkin proteins were subjected to SDS polyacrylamide gel electrophoresis (SDS-PAGE) on 5% stacking gels at 80 V for 30 min and 12% separating gel at 120 V for 60 min. Gels were stained with Coomassie on a shaker for 30 min and destained before visualization.

#### 2.2.3. Evaluating Parkin Activity

Western blot and fluoro-spectrophotometry analysis evaluated the parkin activity. Reactions contained 5 *μ*M of fluorescently-labeled ubiquitin, 15 nM of ubiquitin activase E1, 0.5 *μ*M of ubiquitin-binding enzyme E2, and 1 *μ*M of parkin protein (ubiquitin ligase E3) in 50 mM Tris-HCl (pH 8.0), 2 mM Dithiothreitol (DTT), 5 mM MgCl_2_, 4 mM ATP, and 5% glycerol at 37°C for 1 h. Reactions were terminated through overnight incubation at 4°C and assessed via western blot analysis [15]. Briefly, proteins were separated by SDS-PAGE on polyacrylamide gels (5% stacking gel at 80 V for 30 min and 12% separating gel at 120 V for 60 min) and transferred onto PVDF membranes (Beijing Liuyi Biological Technology Co., Ltd., Beijing, China) at 300 mA for 37 min. Membranes were blocked in 5% BSA in TBS-T (TBS plus 0.1% (*v*/*v*) Tween 20) for 2 h at room temperature with gentle rocking and labeled with the following primary anti-FK-2 antibodies overnight (at 1 : 1000 dilution). Membranes were washed thrice in TBS-T and labeled with horseradish peroxidase- (HRP-) conjugated secondary antibodies (1 : 5000 in 5% BSA/TBS-T) for 1 h at room temperature. Immunoreactive protein bands were visualized using the chemiluminescence system on a ChemiDoc XRS image detector (Jena Analytical Instruments AG, Jena, Germany).

Reactions were analyzed via fluorospectro-photometry by filtering through a 0.5 mL centrifugal filter (Microcon YM-30, Millipore Co., MA, USA) containing a regenerated cellulose membrane with a 30,000 MW cutoff and 14,000 × g centrifugation for 25 min at 4°C. Fluorescence-labeled ubiquitin that did not interact with parkin was discarded. Captured mixtures were washed six times with 200 *μ*L of the reaction buffer at 4°C and centrifuged at 14,000 × g for 25 min to eliminate nonspecific bound fluorescent-labeled ubiquitin. After washing, captured mixtures containing fluorescent-labeled ubiquitin bound to parkin were dissolved in 400 *μ*L of reaction buffer by ultrasonication for 20 min. Finally, the obtained fluorescent solution was measured using a Varian Cary Eclipse fluorescence spectrophotometer (Thermo Fisher Scientific, MA, USA) at 490 nm excitation and 515 nm emission.

### 2.3. Preparation of Analytical Solutions

Reference stock solutions were prepared by dissolving the respective working reference substance in dimethyl sulfoxide (DMSO) to generate 2 mg/mL DL-dithiothreitol (DTT), DL-selenomethionine (ST), amoxicillin (AC), and fenofibrate (FB). A mixed reference solution containing 2 mg/mL of DTT, ST, AC, and FB was prepared in DMSO. Working solutions of PCRR (300 mg/mL) and SFR (400 mg/mL) were prepared by dissolving the freeze-dried powder of the PCRR and SFR extract (in Supplementary Material) in DMSO.

For pharmacological analysis, analytical FB, DTT, K2, K3, K4, H2, and H4 were dissolved in DMSO and diluted in physiological saline to required concentrations. All solutions were stored at 4°C in the dark.

### 2.4. Fishing Parkin Ligands

Analytical solutions (5 *μ*L) containing reference, mixed, PCRR, and SFR working solutions were incubated with parkin suspension (200 *μ*L) at 37°C for 60 min to bind parkin fully. Mixtures were then passed through a 0.5 mL centrifugal filter (Microcon YM-10, Millipore Co., MA, USA) containing a regenerated cellulose membrane with a 10,000 MW cutoff by centrifuging at 14,000 × g for 25 min at 4°C. Parkin/ligand complexes captured in the membranes were washed three times with 200 *μ*L of reaction buffer at 4°C and centrifuged at 14,000 × g for 25 min to eliminate nonspecific binding. Bound ligands were released from the parkin protein by ultrasonic treatment in 80% aqueous methanol solution (400 *μ*L) for 20 min, followed by centrifugation at 14,000 × g for 25 min at room temperature. Ultrafiltrates containing the ligands were then dried under nitrogen flow and redissolved in 100 *μ*L of 80% methanol aqueous solution. Samples were analyzed using LC/MS. The peak area of the experimental samples containing denatured parkin had ≥30% larger Δ*P* values than control samples, suggesting specific binding. Fishing was performed in triplicate and analyzed in duplicate. Δ*P* values were calculated as follows:

(1) $$\begin{array}{c} ∆P>30\%,∆P=\left(Pe–Pc\right)/Pe\times 100 \end{array}$$

*Pc* is the chromatographic peak area of the blank control group, and *Pe* is the chromatographic peak area of the experimental group.

### 2.5. LC/MS Analysis

LC/MS analyses were performed on a UHPLC Dionex Ultimate 3000 system coupled to a Thermo Scientific Q-Exactive TM hybrid quadrupole-orbitrap mass spectrometer with a heated-electrospray ionization probe (Thermo Fisher Scientific, MA, USA). The UHPLC system consisted of a quaternary pump, an autosampler with a temperature control function, a column box, and a photodiode array (PDA) detector. Table S1 shows the UHPLC-PDA conditions.

The HESI-MSn parameters for all samples were as follows: (1) flow rate: 0.2 mL/min (split from HPLC effluent); (2) detection mode: positive and negative ion; (3) heat block and curved desolvation line temperature: 250°C; nebulizing nitrogen gas flow: 1.5 L/min; interface voltage: (+) 3.5 kV, (-) -2.8 kV; (4) mass range: MS, m/z 100-1000; MS2 and MS3, m/z 50-1000; (5) dynamic exclusion time: 10 s; and (6) workstation: Xcalibar 3.0.63 for liquid chromatography combined with data processing, molecular predictions, and precise molecular weight calculations.

### 2.6. Evaluation of Lead Compounds

Parkin autoubiquitination reactions were performed to confirm the ability of the hit compounds to bind to parkin and to evaluate their effects on parkin function. Hit compounds (K2, K3, K4, H2, and H4) were added to a reaction buffer containing 5 *μ*M fluorescently labeled ubiquitin, 15 nM ubiquitin activase E1, 0.5 *μ*M ubiquitin-binding enzyme E2, and 1 *μ*M parkin protein for 1 h at room temperature. Reactions were terminated overnight at 4°C and analyzed by fluoro-spectrophotometry as described (2.2.3).

The 3D structure of the Rattus norvegicus parkin protein molecule (PDB ID: 4k95) [16] was retrieved from the Protein Data Bank (http://www.rcsb.org/). AutodockTools 4 [17] determined the binding affinity of the seven compounds (K1, K2, K3, K4, H1, H2, and H4) toward the full-length autoinhibited parkin protein molecule. The semiflexible docking protocol was followed. The protein molecule was set as rigid and ligands as flexible. The DoGSiteScorer web-server from ProteinsPlus (https://www.proteins.plus/) [18] predicted the binding pocket. A grid box of 96 × 84 × 64 size with 0.416 Å was fixed to cover the Ubl, IBR, Ring1, and REP domains, considering the DoGSiteScorer results and current knowledge on parkin activation. Autogrid4 and autodock4 with Lamarckian genetic algorithms determined the best docking conformations. The PLIP Web Server analyzed protein-ligand interactions [19].

### 2.7. In Vitro Antisteatosis Activity

L02 cells were cultured in RPMI 1640 medium supplemented with 10% fetal bovine serum and 1% penicillin-streptomycin at 37°C in a 5% CO2 incubator. Cells were seeded into 6-well plates at a density of 3 × 10^5^ cells/well and grown to 80-90% confluence. Cells were starved in 0.2% serum for 12 h and exposed to 5% fat emulsion for 24 h. Cells were subsequently treated with kushenol I (25 and 50 *μ*M) and kurarinone (25 and 50 *μ*M) for 24 h and harvested. FB (150 *μ*M) was used as a positive control. Protein concentrations were determined via a BCA assay. TG, TC, AST, ALT, and ATPase levels were determined by the SpectraMax Plus 384 Microplate Reader (Molecular Devices, CA, USA) using commercial diagnostic kits following manufacturers' instructions.

### 2.8. Statistical Analysis

Data were analyzed using IBM SPSS Statistics 21.0 (IBM, NY, USA) and expressed as mean ± SD. A two-tailed Student's *t*-test determined the difference between two groups, while one-way analysis of variance (ANOVA, Dunnett's method) determined the differences between three or more groups. *P* < 0.05 (two-tailed) was considered statistically significant.

## 3. Results and Discussion

### 3.1. Purity and Functional Activity of Purified Parkin

**Figure 1:**
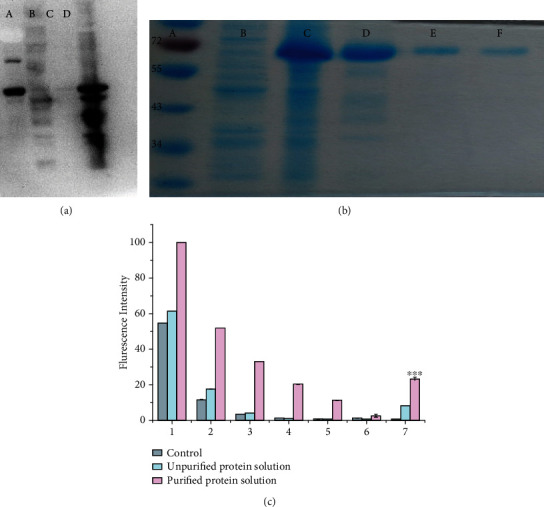
Purity and functional activity of parkin. Coomassie blue staining determined the purity of parkin samples ((a): (A) protein ladder; (B) IPTG; (C) IPTG-induced overexpression; (D) purified with 100 mM imidazole). Western blot analysis of parkin functional activity ((b): (A) protein ladder; (B) unpurified protein; (C) control without parkin; (D) purified proteins) and fluorescence spectrophotometry ((c): 1-6, ultrafiltrates after six washes; 7, solutions containing captured constituents). A one-way analysis of variance (ANOVA) using Dunnett's method determined the differences between groups. ^∗∗∗^*P* < 0.001 compared to the control group under identical conditions.

The purification procedure produced more high-quality parkin with low miscellaneous proteins than unpurified samples, suggesting high levels of parkin enrichment (Figure 1(a)). The purified parkin protein solution had several FK2 reactive bands than the crude prep, suggesting higher purity and biological activity (Figure 1(b)). FK2 antibodies recognize mono-ubiquitinated and polyubiquitin proteins [15].

Ubiquitin (~8.5 kDa) covalently binds to parkin (~52 kDa) during its ubiquitination, which can be captured using ultrafiltration membranes at 30 kDa molecular weight (MW) cutoff. Centrifugation discards unbound ubiquitin that penetrates the ultrafiltration membrane. The fluorescence intensity of ultrafiltrates containing fluorescence-labeled ubiquitin declined with increasing washes, with a near-complete loss of fluorescence at the 6th washing stage, suggesting the removal of noncovalently bound ubiquitin proteins (Figure 1(c)). The fluorescence intensity of the experimental solution containing purified parkin was significantly higher than crude and parkin negative samples. These results suggest that the purified parkin was ubiquitinated and showed biological activity.

### 3.2. Reliability of the Parkin-Ligand Fishing Method

The reliability of the method was evaluated using ST and DTT as positive controls and AC and FB as negative controls. Mixed reference solutions of these compounds were also assessed.

The reproducibility of the fishing method was first examined using ST and DTT. Variations were expressed as the relative reference deviation (RSD) of the peak area of the compound. The RSDs (*n* = 3) for ST and DTT were 15.90 and 20.13%, respectively, indicating that the procedure was precise for qualitatively evaluating parkin ligands.

**Figure 2:**
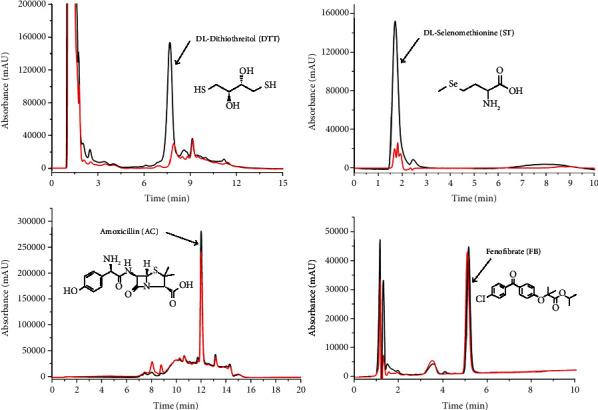
Analysis of four reference solutions using the parkin-based fishing method. HPLC chromatograms of ultrafiltrates containing references released from active (black line) or denatured parkin (red line). Enhancement of the peak area of the references contrasting control samples indicated specific parkin binding.

Next, the ST, DTT, AC, and FB reference and mixed solutions evaluated the recognition, separation, and identification capability of the fishing method. Denatured parkin was included as a control (red line). Reference solutions were independently determined using the fishing method.  Figure 2 shows HPLC chromatograms. The ST and DTT peaks showed prominent areas of enhancement than the controls containing denatured parkin (∆*P* values shown in Table S2 were 37.1–56.3%), indicating specific binding to active parkin. However, the AC and FB peak areas were nearly identical to the controls (∆*P* < 30%, shown in Table S2). Mass spectrometry data (Table S2) confirmed that the peaks were ST, DTT, AC, and FB. ST and DTT were confirmed as specific parkin binders.

**Figure 3:**
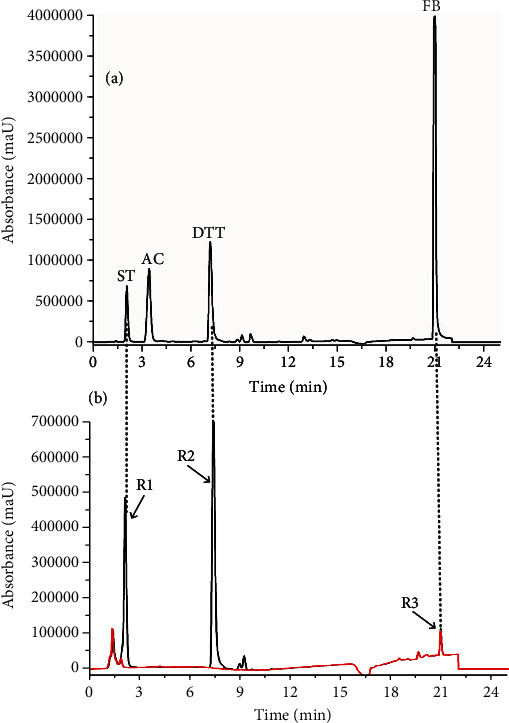
Determination of a mixed reference solution using the parkin-based fishing method. (a) LC/MS directly assayed HPLC chromatograms of a mixed reference solution. (b) HPLC chromatograms of a mixed reference solution for ultrafiltrates derived from active (black line) and denatured parkin (red line). Peaks R1 and R3 exhibited area enhancement than the controls. ST: DL-selenomethionine; AC: amoxicillin; DTT: DL-dithiothreitol; FB: fenofibrate.

Mixed reference solutions were analyzed and detected by LC/MS.  Figure 3 shows HPLC chromatograms. The R1 (∆*P* = 37.1 ± 12.2%, *n* = 3) and R2 (∆*P* = 56.3 ± 15.5%, *n* = 3) peaks were enhanced than the controls, showing specific parkin binding. The R3 peak was nearly identical to the control samples (∆*P* = 6.1 ± 7.4%, *n* = 3). Comparing the retention times of the peaks between the two chromatograms (Figures 3(a) and 3(b)) showed that the R1, R2, and R3 peaks were DTT, ST, and FB, respectively. AC was not detected during the fishing procedure. The fishing method detected ST and DTT that specifically bind parkin.

ST and DTT displayed specific binding to active parkin with ∆*P* > 30%, while AC and FB showed minimal binding (∆*P* < 30%). Therefore, peaks with ∆*P* > 30% indicated the presence of parkin-specific ligands. DTT [20] and ST (refer to the PDB database) are known parkin interactors. In contrast, AC [21], a *β*-lactam antibiotic that inhibits the synthesis of bacterial cell walls, does not bind to parkin but selectively interacts with penicillin-binding proteins abundant in bacteria. FB [22] shows minimal binding to parkin but selectively activates PPAR-*α* and PPAR-*γ*. Both AC and FB did not interact with parkin in our assays, highlighting the selective recognition, separation, and identification potential of the method.

### 3.3. Influence of Assay Conditions

Parkin ligands in the SFR and PCRR extracts were screened using the fishing method under various reaction conditions. Parkin concentrations, sample concentrations, and incubation times were varied to investigate optimal assay parameters. At increasing parkin concentrations (0.25, 0.50, and 1.0 g/L), the sensitivity of the assay increased, and the number of parkin ligands detected from HMs was more abundant (Figures S2 and S5). When the concentration of parkin for SFR and PCRR decreased to 0.50 g/L, the number of ligands in the extracts decreased. We, therefore, selected 1.0 g/L parkin for subsequent assays as higher concentrations will compromise the ultrafiltration membrane.

Sample concentration also influenced the ability to screen parkin ligands. In complex samples of HMs, active components have low abundances and are undetectable by LC/MS. However, increasing sample concentration increases the interference of nonactive components and the probability of false positives. This study investigated three concentrations of PCRR (1.875, 3.750, and 7.500 g/L) and SFR (2.625, 5.25, and 10.5 g/L). The number of parkin ligands identified from PCRR and SFR increased with increasing sample concentration (Figure S3 and S6). Nevertheless, PCRR (7.500 g/L) and SFR (10.5 g/L) sample concentrations were optimal.

Incubation times also influenced the screening assays. Short incubation periods prevent identifying target molecules not bound to parkin, while longer incubation times structurally change the binding compound. Therefore, 30, 60, and 90 min incubation times were assessed for both PCRR and SFR samples. The results revealed that 60 min was optimal for the screening SFR extracts (Figures S4) and PCRR (Figures S7).

### 3.4. Fishing of Parkin Ligands from SFR and PCRR

**Figure 4:**
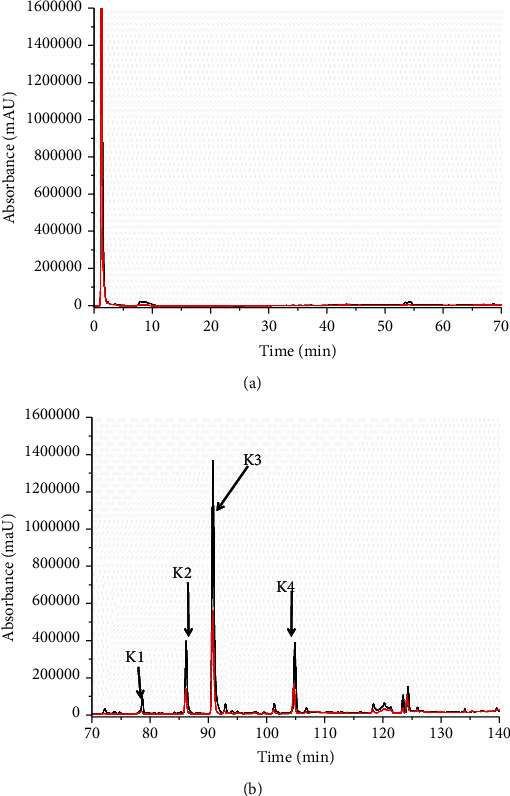
Fishing of parkin-targeted constituents from SFR extracts. HPLC chromatograms of screened SFR extract ((a, b): (a) 0–70 min; (b) 70–140 min) showing four peaks (K1–K4) that were significantly enhanced by specific parkin binding (black line) against controls containing denatured parkin (red line).

**Figure 5:**
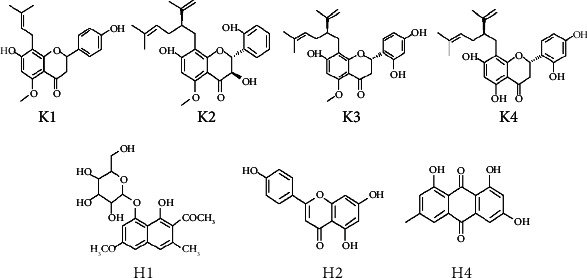
Chemical structures of the hit compounds fished from SFR and PCRR extracts. K1: 4′-dihydroxy-5-methoxy-8-(*γ*,*γ*-dimethylallyl)-flavanone; K2: kushenol I; K3: kurarinone; K4: sophoraflavanone G; H1: torachrysone-8-O-glucoside; H2: apigenin; H4: emodin.

**Figure 6:**
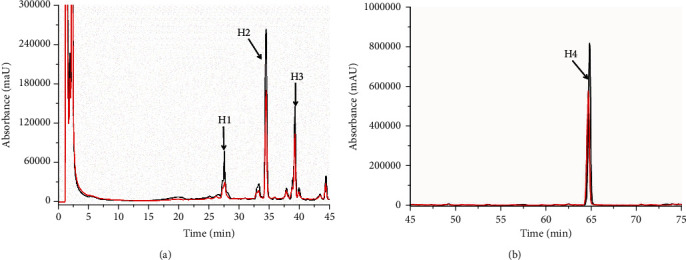
Fishing of parkin-targeted constituents from PCRR extracts. HPLC chromatograms of screened PCRR extracts ((a, b): (a) 0–45 min; (b) 45–75 min) showing four peaks (H1–H4) that were significantly enhanced due to specific parkin binding (black line) against denatured parkin (red line).

The described method was designed to identify parkin ligands in SFR and PCRR extracts.  Figure 4 shows chromatograms of the analyzed SFR sample solution. The chromatograms revealed significant enhancement of four peaks (K1–K4) compared to denatured parkin (∆*P* > 30%, Table S3), indicating specific parkin binding. The UV, MS, and MSn analysis of the LC/MS data (Table S3), and comparison to previous studies [23, 24] and references, confirmed the K1–K4 peaks as 7,4′-dihydroxy-5-methoxy-8-(*γ*, *γ*-dimethylallyl)-flavanone (K1), kushenol I (K2), kurarinone (K3), and sophoraflavanone G (K4), respectively (Figure 5). Analyzing the PCRR sample (Figure 6) confirmed four peaks (H1–H4) (∆*P* > 30%, Table S4), representing the active parkin constituents. Characterizing the UV, MS, and MSn data obtained by LC/MS (Table S4) and comparison with previous data [25–27] and references verified the H1, H2, and H4 peaks as torachrysone-8-O-glucoside (H1), apigenin (H2), and emodin (H4), which are flavones, naphthalene, and anthraquinones, respectively (Figure 5). These compounds represent previously undescribed, novel parkin interacting proteins.

### 3.5. Effects of Hit Compounds on Parkin Activity

**Figure 7:**
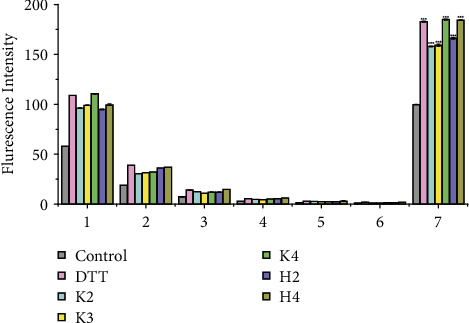
Effects of the hit compounds on parkin activity. A one-way analysis of variance (ANOVA) following Dunnett's method evaluated significant differences between groups. ^∗∗∗^*P* < 0.001 compared with the control group under identical conditions. 1-6: ultrafiltrates after six washes; 7: solutions containing captured constituents; DTT: DL-dithiothreitol; K2: kushenol I; K3: kurarinone; K4: sophoraflavanone G; H2: apigenin; H4: emodin.

The fluorescence intensity of the ultrafiltrates containing fluorescence-labeled ubiquitin weakened with washing and was near-absent at the sixth washing stage (Figure 7). This pattern suggested the successful removal of noncovalently bound ubiquitin proteins from the reaction solution. In contrast, the fluorescence intensity of the solution containing captured constituents from DTT, K2, K3, K4, H2, and H4 groups was significantly enhanced than the control group. This enhanced intensity indicated that the hit compounds enhanced parkin activity and parkin-mediated ubiquitination.

**Figure 8:**
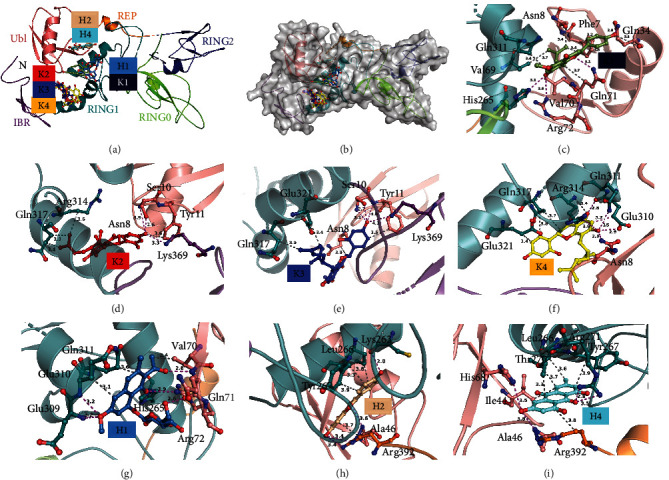
Docking analysis of parkin (PDB ID: 4K95) with the seven hit compounds. (a, b) Overall presentations of the binding sites of the seven hit compounds. The parkin protein (a) and surface (b) modes. The ball-and-stick modes with different colors represent the compounds. Different colors illustrate the parkin Ubl, RING0, RING1, IBR, REP, and RING2 domains. (c–i) Close-up views of the seven compounds interacting with parkin. The protein was shown in cartoon mode, while the compounds and coordinated amino acids were shown in ball-and-stick mode. The purple dashed lines with distances (Å) represent hydrogen bonds, while gray dashed lines represent hydrophobic interactions.

Molecular docking elucidated the potential interactions between the hit compounds and parkin protein. The seven hit compounds K1, K2, K3, K4, H1, H2, and H4 bound to the parkin protein at -6.5 kcal/mol, -6.24 kcal/mol, -6.11 kcal/mol, -6.15 kcal/mol, -5.24 kcal/mol, -7.38 kcal/mol, and -7.63, respectively, in their best conformations (Table 1). H1 and K1 share a binding pocket in RING1 and UBI domains, while H2 and H4 bind in a pocket at the other side of these two domains (Figures 8(a) and 8(b)). K2, K3, and K4 bind in a similar pocket with amino acids from the IBR and RING1 domains. All the complexes commonly have hydrogen bonds and hydrophobic interactions, while H1-4k95 and H2-4K95 complexes had a salt bridge or pi-cation interactions, respectively (Figures 8(c)–8(i)). The interactions mainly occurred with amino acids of the RING1 and Ubl domains. A stable interaction of the RING1 and Ubl domains directly causes the native parkin autoinhibited state [28, 29]. Thus, compounds binding to this area may disrupt this conformation, consequently activating parkin. The cocrystallization strategy may accurately address the mechanism of parkin activation by the hit compounds. Five of the tested compounds were shown to be parkin ligands, highlighting the reliability of the developed fishing method. Compounds K1–K4 and H1–H4 directly act on parkin and may represent the bioactive constituents of SFR and PCRR, respectively. Thus, these constituents may help treat parkin-related diseases, including cancer, neurodegenerative disorders, liver, and heart diseases.

### 3.6. Antisteatosis Activity of the Hit Compounds

**Figure 9:**
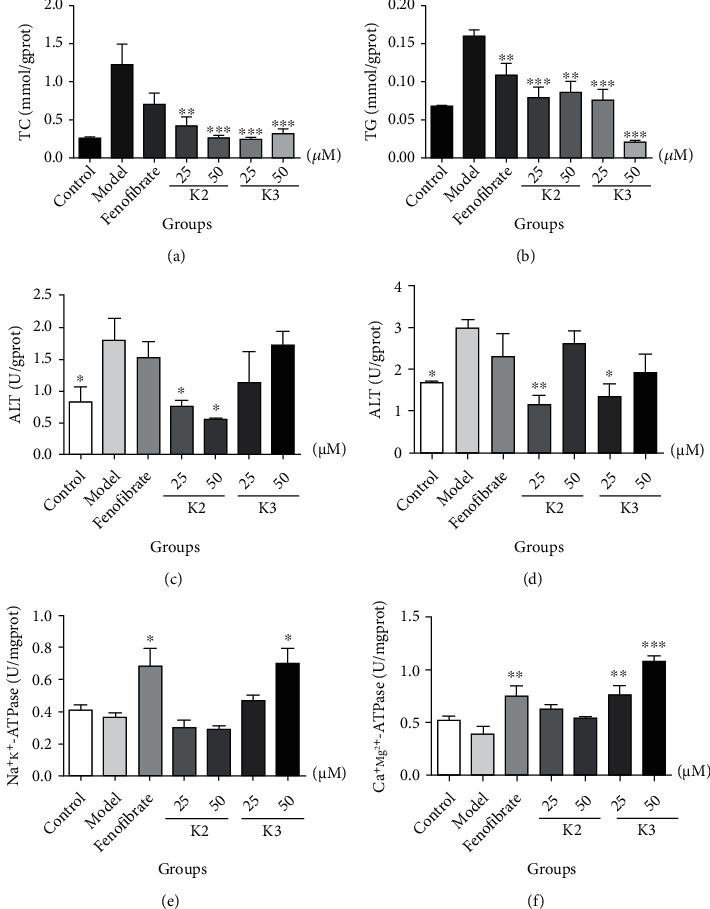
Effects of the hit compounds on fat emulsion induced L02 adipocytes. Significant differences between groups were evaluated using a one-way analysis of variance (ANOVA) using Dunnett's method (*n* = 3). ^∗^*P* < 0.05, ^∗∗^*P* < 0.01, ^∗∗∗^*P* < 0.001. K2: kushenol I; K3: kurarinone.

Fat emulsion treatment significantly increased TC, TG, ALT, and AST levels in L02 cells, while Na^+^-K^+^-ATPase and Ca^2+^-Mg^2+^-ATPase levels remained unaffected (Figure 9). K2 and K3 treatments restored TC, TG, ALT, and AST levels, while the levels of Na^+^-K^+^-ATPase and Ca^2+^-Mg^2+^-ATPase significantly increased. This fishing analysis deduced that K2 and K3 directly bind and regulate parkin activity, preventing hepatocyte steatosis and highlighting the K2 and K3 potential for treating fatty liver disease. Therefore, the developed fishing method represents an effective alternative for discovering lead compounds and drugs from HMs.

## 4. Conclusions

We developed an efficient method to systematically fish parkin ligands from complex matrices, including PCRR and SFR. The method exhibited excellent recognition, separation, and identification and was validated using positive and negative controls. The method was fast, simple, and required minimal training or sample preparation. We successfully identified seven parkin ligands from PCRR and SFR extracts and directly confirmed the regulatory activity of five compounds on parkin using *in vitro* autoubiquitination assays and molecular docking analysis. Cell-based trials showed the antisteatosis activity of two hit compounds (kushenol I and kurarinone). Therefore, the developed method efficiently isolated parkin ligands in complex systems and may elucidate the mechanism(s) of drug activity and the development of new HM drugs.

## Figures and Tables

**Table 1:** Molecular docking of the seven hit compounds to the parkin protein molecule (PDB ID: 4K95).

No.	Name of compound	Binding energy kcal/mol	Interacting residues
K1	7,4′-Dihydroxy-5-methoxy-8-(*γ*,*γ*-dimethylallyl)-flavanone	-6.5	Phe7, Asn8, *Gln34*, Val70, *Gln71*, *Arg72*, *His265*, Val269, Gln311
K2	Kushenol I	-6.24	*Ser10*, Tyr11, Arg314, Gln317, *Lys369*
K3	Kurarinone	-6.11	Val70, *Gln71*, *Arg72*, *His265*, *Glu309*, *Gu310*, Gln311
K4	Sophoraflavanone G	-6.15	Asn8, *Gu310*, *Gln311*, *Arg314*, Gln317, Glu321
H1	Torachrysone-8-O-glucoside	-5.24	Val70, *Gln71*, *Arg72*, *His265*, *Glu309*, *Gu310*
H2	Apigenin	-7.38	*Ala46*, *Cys263*, Leu266, Leu266, Try267, *Arg392*
H4	Emodin	-7.63	Ile44, *Ala46*, *His68*, Leu266, Try267, Thr270, *Arg271*, Arg392

The italic amino acids indicate hydrogen bonds.

## Data Availability

The data used to support the findings of this study are available from the corresponding author upon request.

## Additional Points

*Highlights*. (i) An efficient method to capture parkin ligands from herbal medicines is presented. (ii) Seven novel parkin ligands are identified. (iii) The antisteatosis activity of two parkin ligands is confirmed.

## Supplementary Materials

Supplementary Materials Supplementary Experimental Procedures. Supplementary Figures and Tables. Figure S1: analytical procedures for the identification of parkin ligands in 24 herbal medicines. Figure S2: effect of parkin concentration on fishing of parkin ligands from SFR extract. Figure S3: effect of concentration of SFR sample on fishing of parkin ligands. Figure S4: effect of incubation time on fishing of parkin ligands from SFR extract. Figure S5: effect of parkin concentration on fishing of parkin ligands from PCRR extract. Figure S6: effect of concentration of PCRR sample on fishing of parkin ligands. Figure S7: effect of incubation time on fishing of parkin ligands from PCRR extract. Table S1: HPLC–DAD conditions for all analyzed samples. Table S2: LC/MS data for the four reference solutions. Table S3: LC/MS data and assignment of four parkin ligands in SFR extract. Table S4: LC/MS data and assignment of four parkin ligands in PCRR extract.
