# Supplementary material for: A Novel Method for Identifying Parkin Binding Agents in Complex Preparations of Herbal Medicines
Source: Oxid Med Cell Longev. 2022 Jan 18;2022:3260243. doi: 10.1155/2022/3260243 (PMC8789414; doi:10.1155/2022/3260243)
Supplement: Supplementary Materials — Supplementary Experimental Procedures. Supplementary Figures and Tables. Figure S1: analytical procedures for the identification of parkin ligands in 24 herbal medicines. Figure S2: effect of parkin concentration on fishing of parkin ligands from SFR extract. Figure S3: effect of concentration of SFR sample on fishing of parkin ligands. Figure S4: effect of incubation time on fishing of parkin ligands from SFR extract. Figure S5: effect of parkin concentration on fishing of parkin ligands from PCRR extract. Figure S6: effect of concentration of PCRR sample on fishing of parkin ligands. Figure S7: effect of incubation time on fishing of parkin ligands from PCRR extract. Table S1: HPLC–DAD conditions for all analyzed samples. Table S2: LC/MS data for the four reference solutions. Table S3: LC/MS data and assignment of four parkin ligands in SFR extract. Table S4: LC/MS data and assignment of four parkin ligands in PCRR extract. [file 3260243.f1.pdf]

SUPPLEMENTARY INFORMATION

A Novel Method for Identifying Parkin Binding Agents in Complex Preparations of Herbal Medicines

Feng-Jiao Li [a,b,#], Fan Zhang [a,d,#], Xu-Dong He [a,d,#], Xin Liu[c], Jian-Kang Mu [a,d], Min Yang [a,d], Yan-Qin Li [a,d], Wen Gu [a,d], Jie Yu [a,d]*, Xing-Xin Yang [a,d,]*

[a] College of Pharmaceutical Science, Yunnan University of Chinese Medicine, 1076 Yuhua Road, Kunming 650500, China

[b] Zhaotong City Institute of Gastrodia Elata, Zhaotong 657000, China

[c] Beijing Entry-Exit Inspection and Quarantine Bureau, Beijing 100026, China

[d] Yunnan Key Laboratory of Southern Medicine Utilization, 1076 Yuhua Road, Kunming 650500, China

[#] These authors contributed equally to this work

Corresponding author: Dr. Jie Yu (Tel./Fax.: +86-871-65933303; Email: cz.yujie@gmail.com) and Dr. Xing-Xin Yang (Tel./Fax.: +86-871-65933303; Email: yxx78945@163.com)

Table of contents

Supplementary Experimental Procedures

Supplementary Figures and Tables

1. **FIGURE S1.** Analytical procedures for the identification of Parkin ligands in herbal medicines.
2. **FIGURE S2.** Effect of Parkin concentration on fishing of Parkin ligands from SFR extract.
3. **FIGURE S3.** Effect of concentration of SFR sample on fishing of Parkin ligands.
4. **FIGURE S4.** Effect of incubation time on fishing of Parkin ligands from SFR extract.
5. **FIGURE S5.** Effect of Parkin concentration on fishing of Parkin ligands from PCRR extract.
6. **FIGURE S6.** Effect of concentration of PCRR sample on fishing of Parkin ligands.
7. **FIGURE S7.** Effect of incubation time on fishing of Parkin ligands from PCRR extract.
8. **Table S1.** HPLC–DAD conditions for all analyzed samples.
9. **Table S2.** LC/MS data for the four reference solutions.
10. **Table S3.** LC/MS data and assignment of four Parkin ligands in SFR extract.
11. **Table S4.** LC/MS data and assignment of four Parkin ligands in PCRR extract.

Supplementary Experimental Procedure

Preparation of freeze-dried powder of Sophorae Flavescentis Radix (SFR) and Polygoni Cuspidati Rhixoma et Radix (PCRR) extract.

Pulverized dried samples of SFR and PCRR (50 g) were soaked for 30 min in 500 mL methanol, and extracted using ultrasonication (500 W, 40 KHz) for 60 min. The extracted solution was filtered and collected, and the residue was ultrasonicated (500 W, 40 KHz) with 400 mL of 70% methanol for another 60 min. Extracted solutions were then filtered. Next, the two successive filtrates were mixed and evaporated using an N-1100D-WD rotatory evaporator (Ai Lang Instrument Co., Ltd., Shanghai, China) at 45 ˚C under reduced pressure. Finally, the concentrate was lyophilized to powder with a FD8-10B freeze dryer (SIM International Group Co. Ltd., Newark, DE, USA). Obtained powder of SFR and PCRR extracts was stored in a dryer at room temperature until use.

Supplementary Figures and Tables

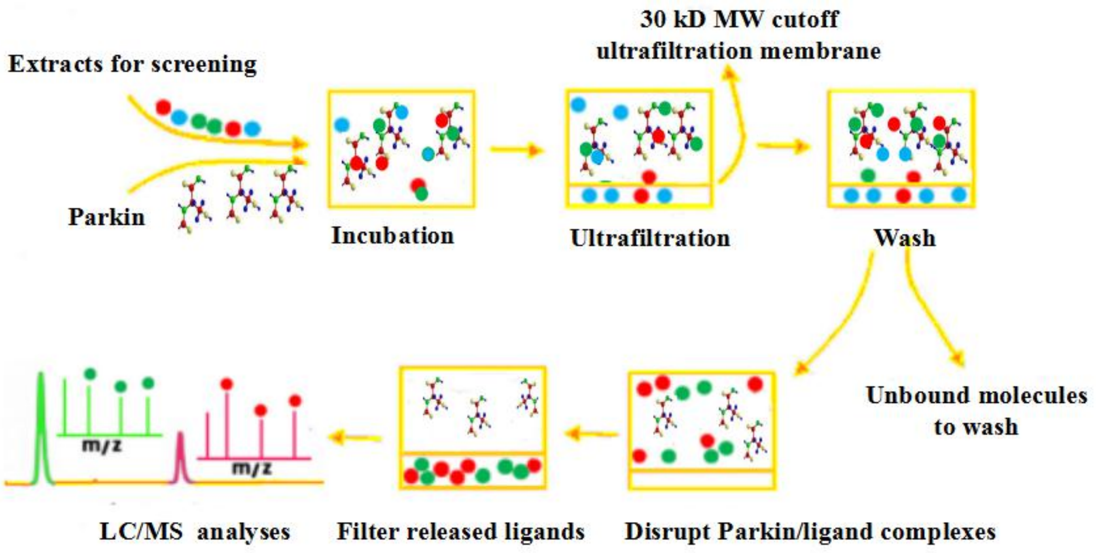

FIGURE S1. Analytical procedures for the identification of Parkin ligands in herbal medicines.

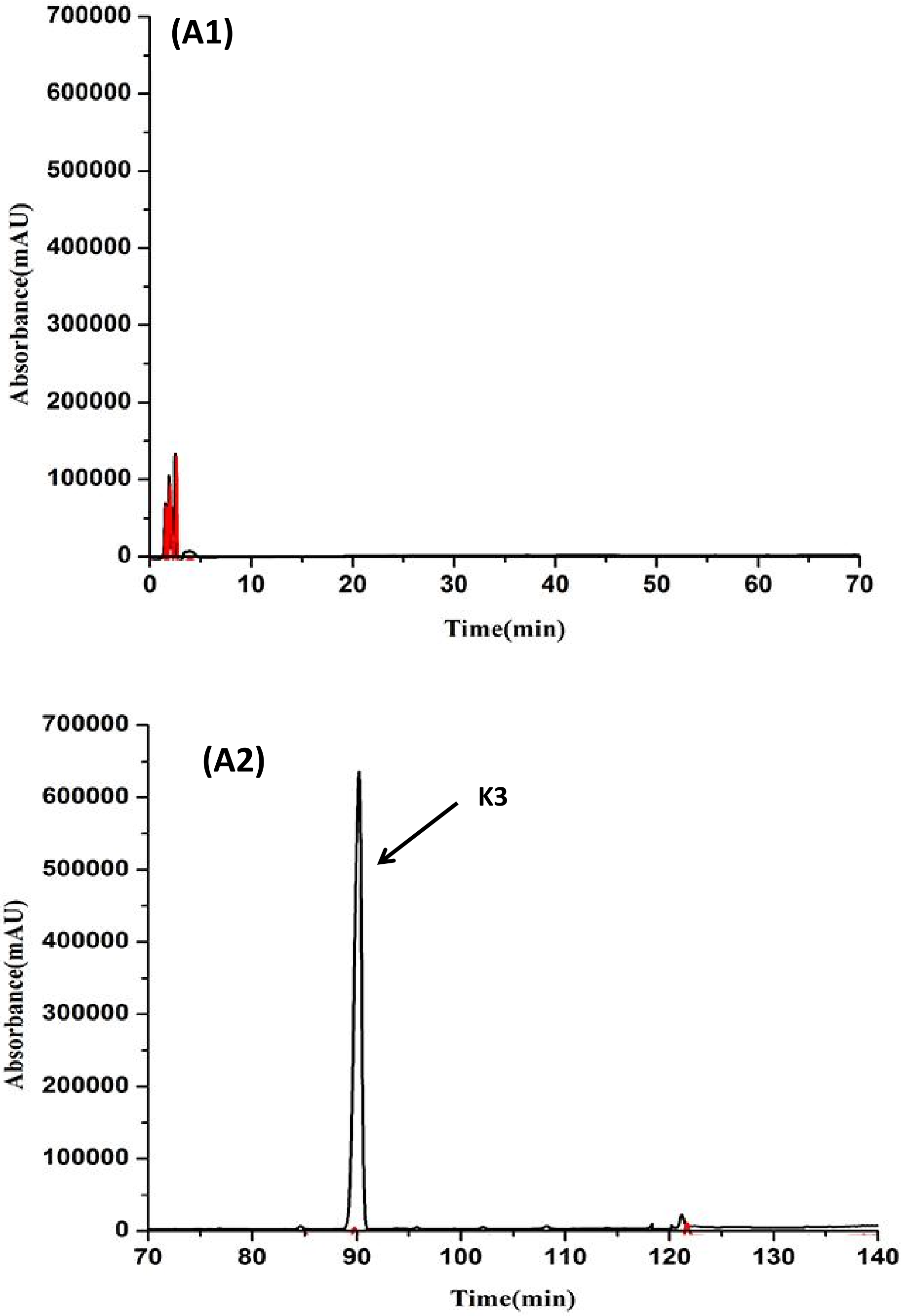
(A1)
Absorbance(mAU)
Time(min)
(A2)
K3
Absorbance(mAU)
Time(min)

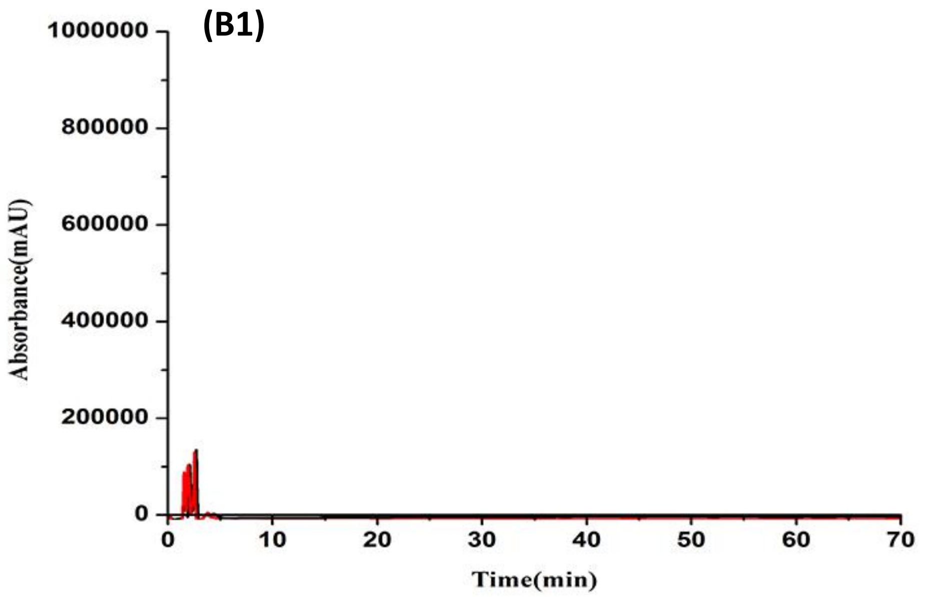
(B1)
1000000
800000
600000
400000
200000
0
Absorbance(mAU)
0
10
20
30
40
50
60
70
Time(min)

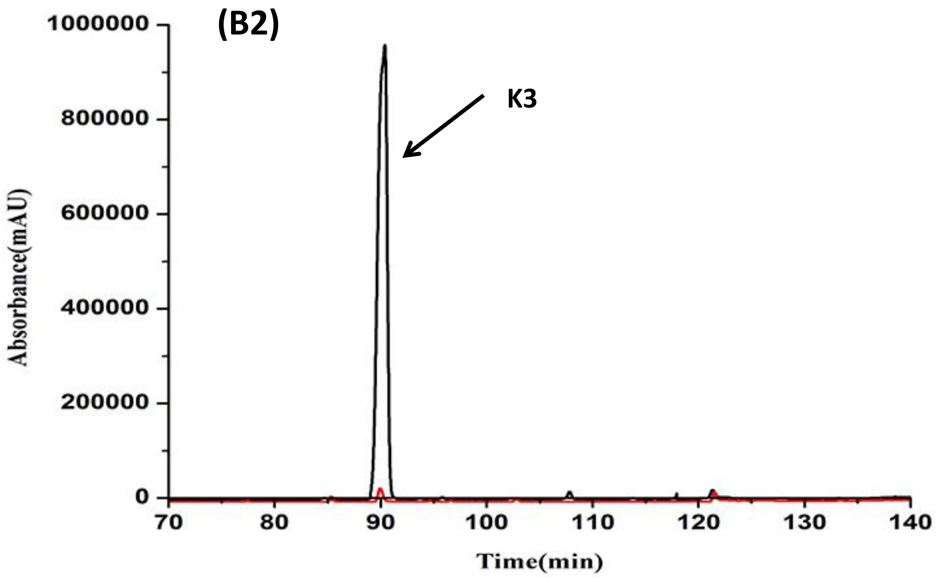
(B2)
K3
1000000
800000
600000
400000
200000
0
Absorbance(mAU)
70
80
90
100
110
120
130
140
Time(min)

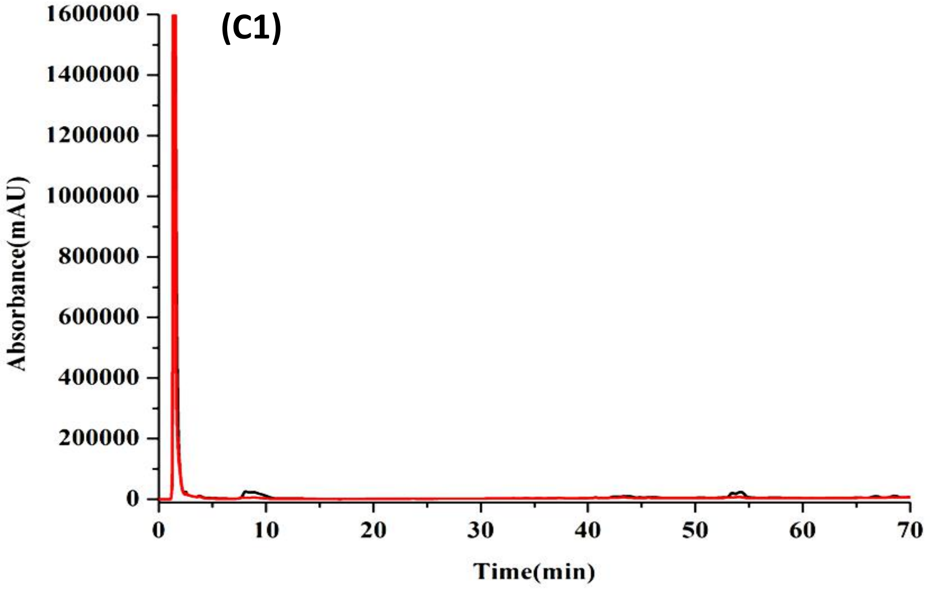

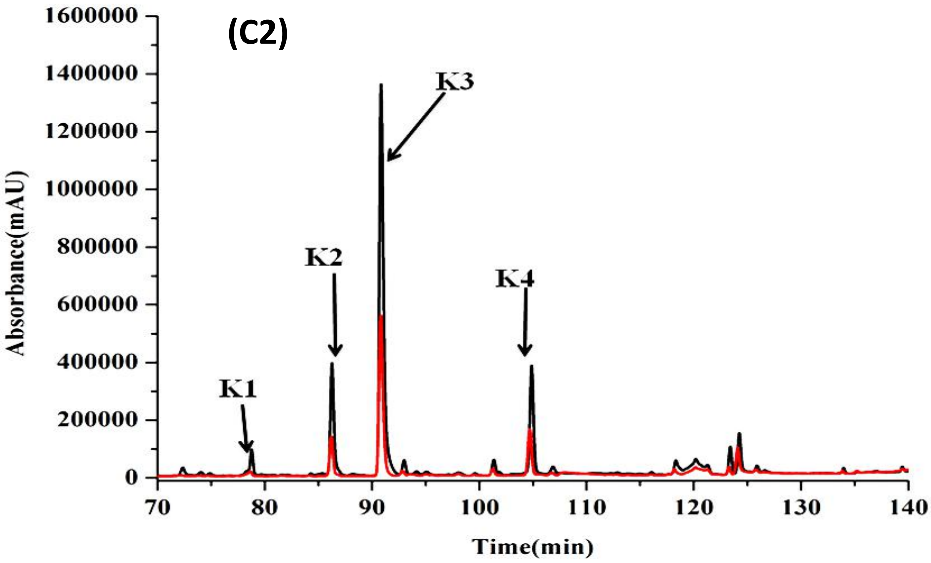

FIGURE S2. Effect of Parkin concentration on fishing of Parkin ligands from SFR extract. The concentrations of Parkin were 0.25 (A1 and A2; A1, 0–70 min; A2, 70–140 min), 0.50 (B1 and B2; B1, 0–70 min; B2, 70–140 min) and 1.00 (C1 and C2; C1, 0–70 min; C2, 70–140 min). Compared to the control, which was comprised of denatured Parkin (red line), the HPLC chromatograms of the screened SFR extract showed four (K1–K4) peaks that were enhanced due to specific binding with Parkin (black line). The concentration of SFR sample and incubation time were 10.5 g/L and 60 min, respectively.

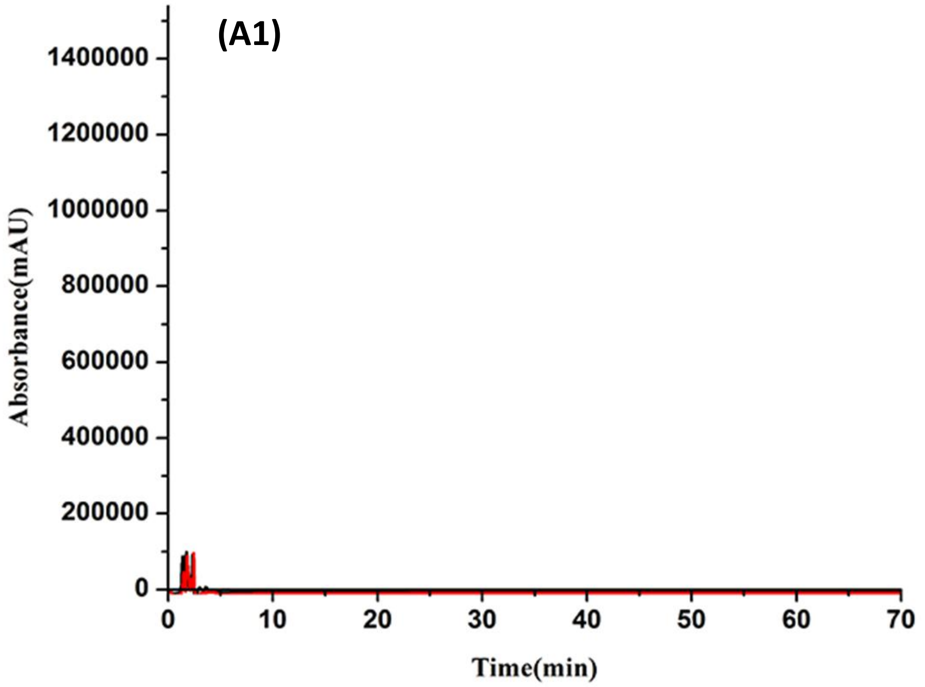
(A1)
Absorbance(mAU)
1400000
1200000
1000000
800000
600000
400000
200000
0
0
10
20
30
40
50
60
70
Time(min)

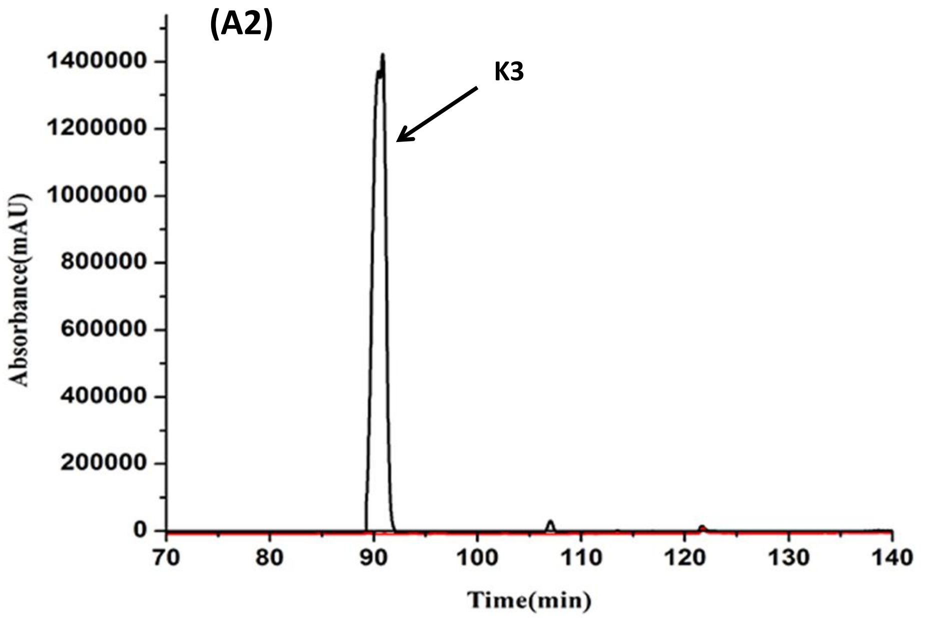
(A2)
K3
Absorbance(mAU)
1400000
1200000
1000000
800000
600000
400000
200000
0
70
80
90
100
110
120
130
140
Time(min)

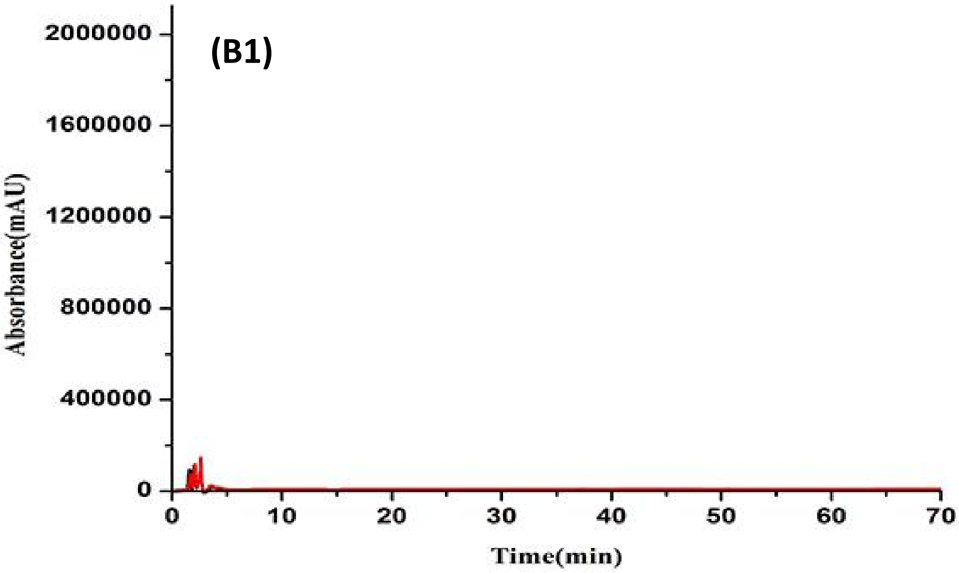
(B1)
Absorbance(mAU)
2000000
1600000
1200000
800000
400000
0
0
10
20
30
40
50
60
70
Time(min)

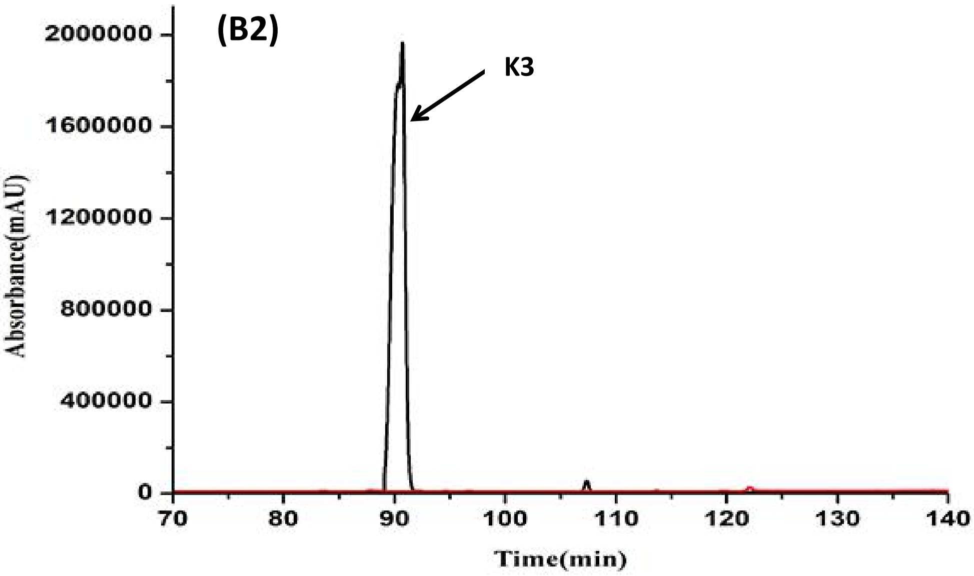
(B2)
K3
Absorbance(mAU)
2000000
1600000
1200000
800000
400000
0
70
80
90
100
110
120
130
140
Time(min)

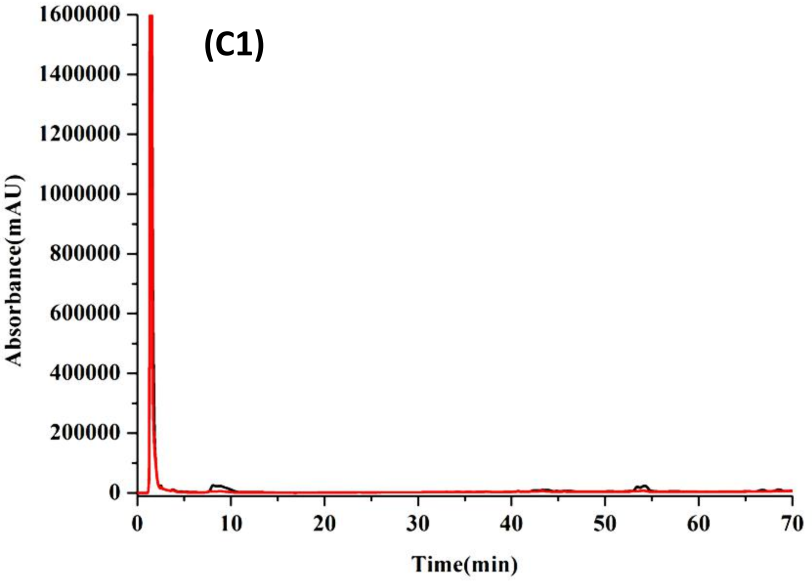

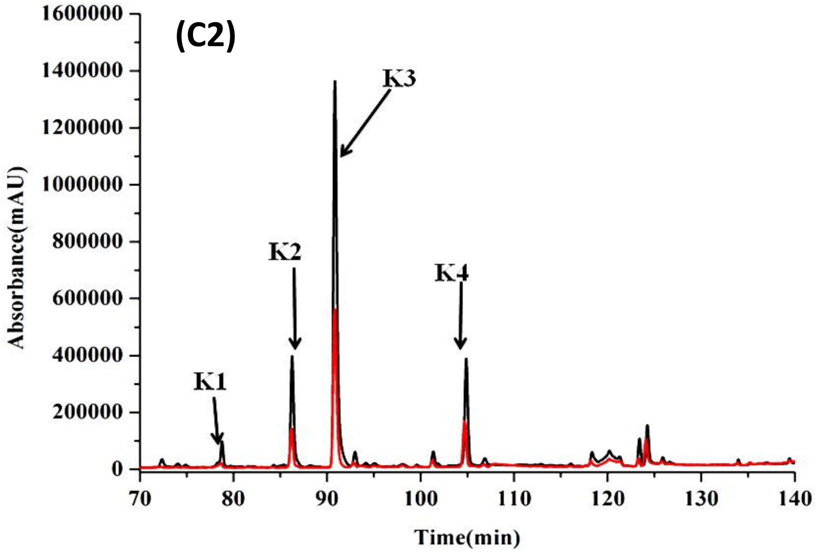

FIGURE S3. Effect of concentration of SFR sample on fishing of Parkin ligands. The concentrations of SFR sample were 2.625 (A1 and A2; A1, 0–70 min; A2, 70–140 min), 5.25 (B1 and B2; B1, 0–70 min; B2, 70–140 min) and 10.50 (C1 and C2; C1, 0–70 min; C2, 70–140 min). Compared to the control, which was comprised of denatured Parkin (red line), the HPLC chromatograms of the screened SFR extract showed four (K1–K4) peaks that were enhanced due to specific binding with Parkin (black line). The concentration of Parkin sample and incubation time were 1.0 g/L and 60 min, respectively.

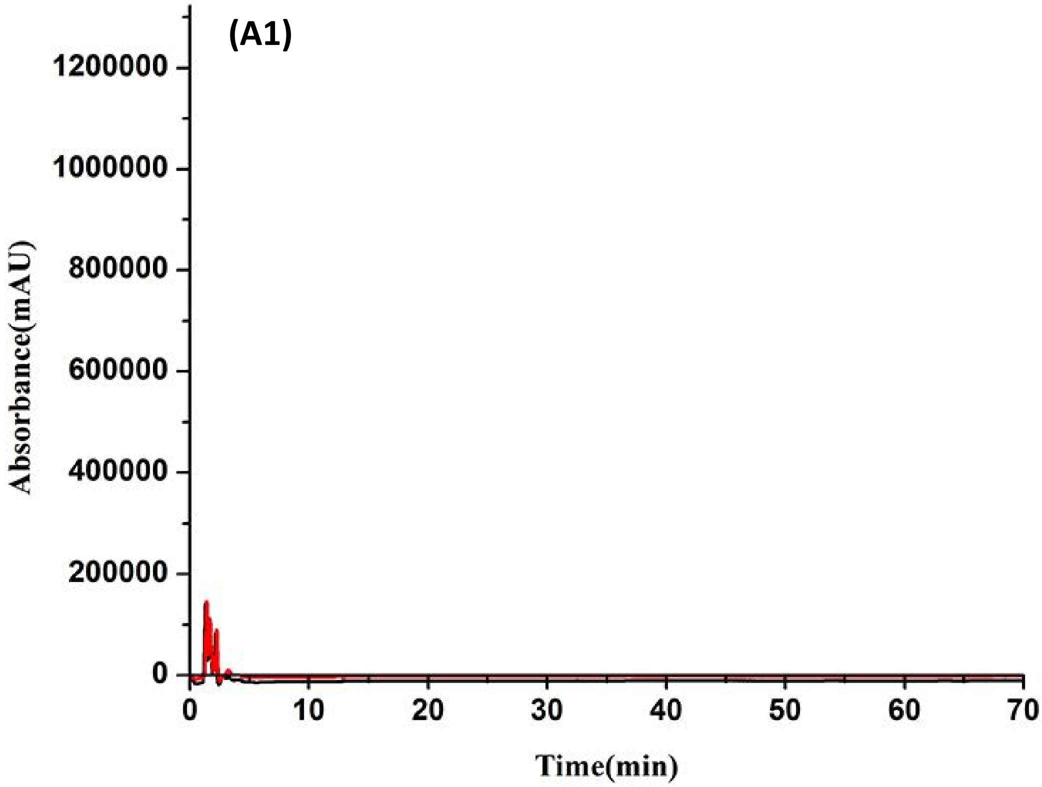
(A1)
Absorbance(mAU)
Time(min)
0
200000
400000
600000
800000
1000000
1200000
0
10
20
30
40
50
60
70

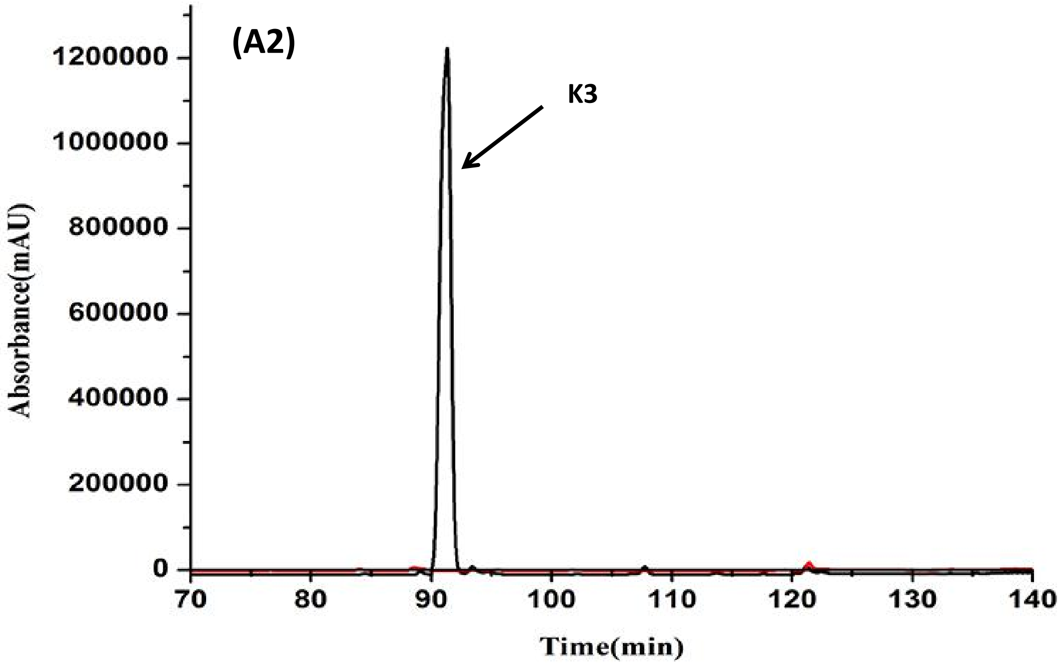
(A2)
K3
Absorbance(mAU)
Time(min)
0
200000
400000
600000
800000
1000000
1200000
70
80
90
100
110
120
130
140

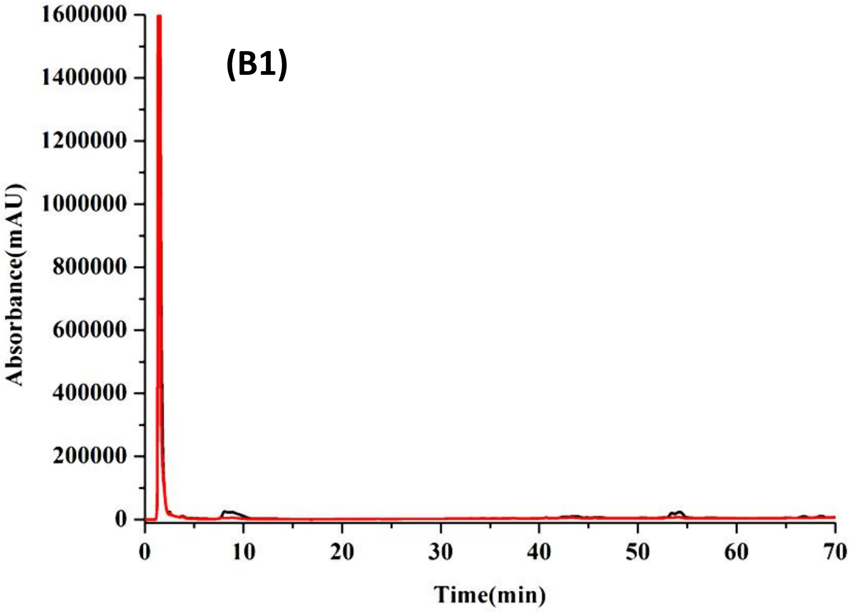
(B1)
Absorbance(mAU)
Time(min)
1600000
1400000
1200000
1000000
800000
600000
400000
200000
0
0
10
20
30
40
50
60
70

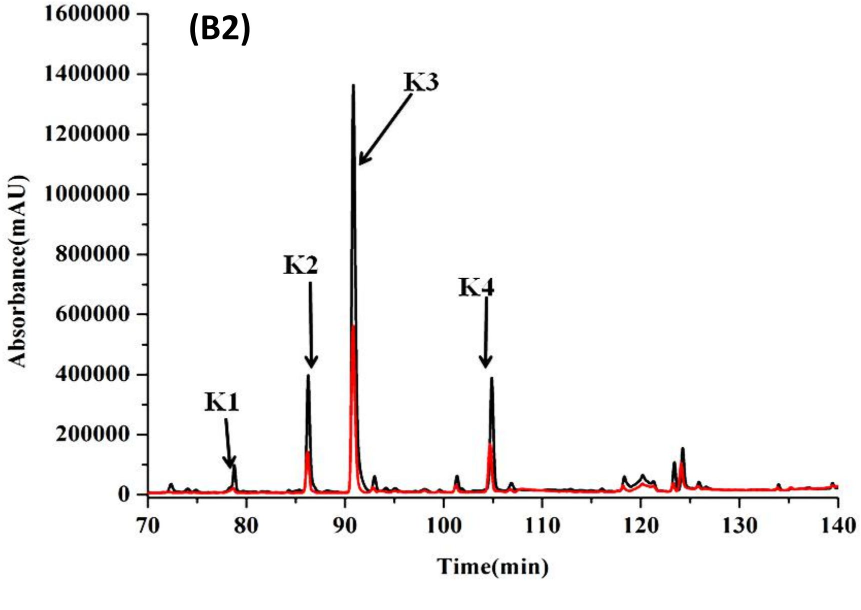
(B2)
K1
K2
K3
K4
Absorbance(mAU)
Time(min)
1600000
1400000
1200000
1000000
800000
600000
400000
200000
0
70
80
90
100
110
120
130
140

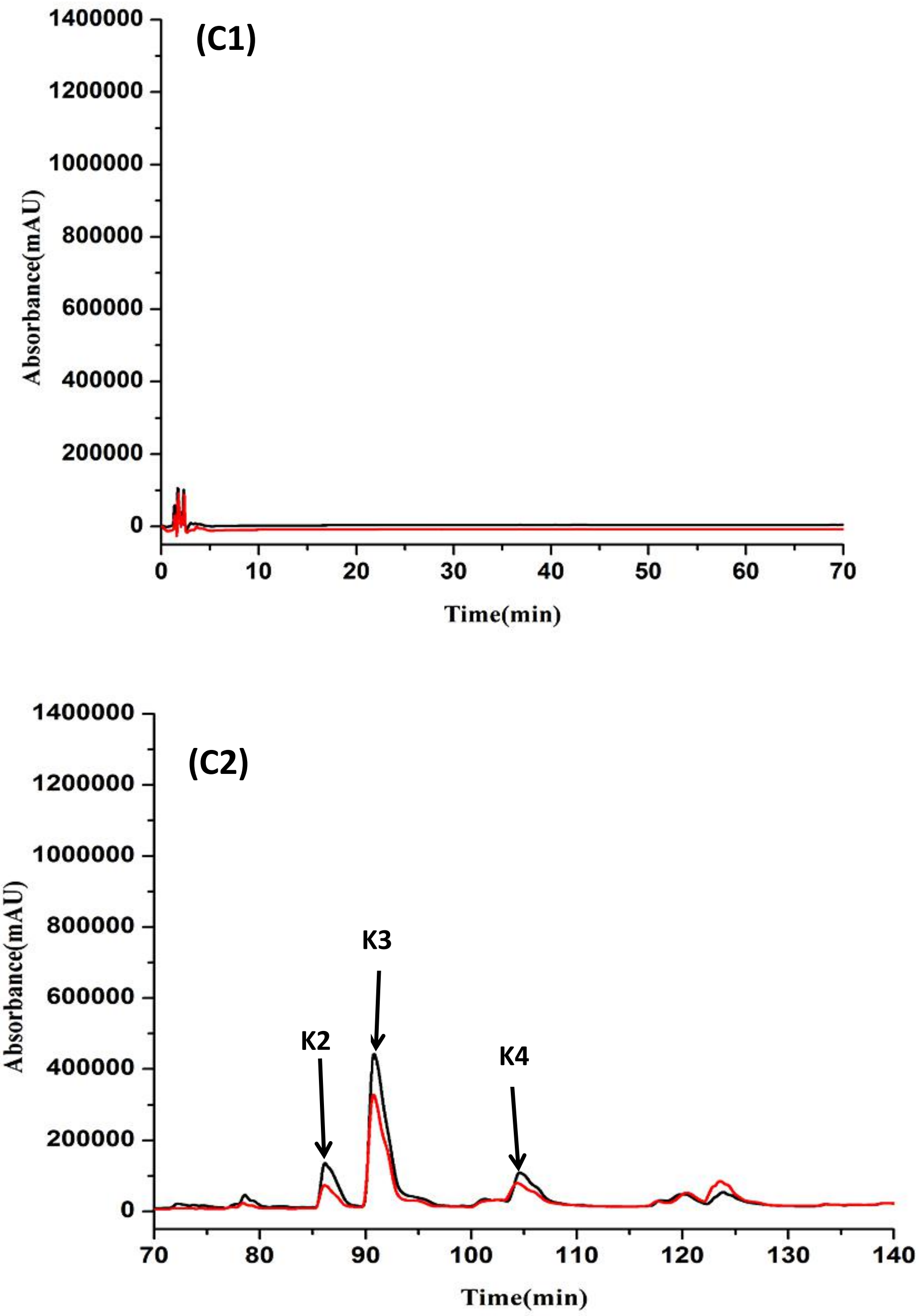

FIGURE S4. Effect of incubation time on fishing of Parkin ligands from SFR extract. Incubation times were 30 (A1 and A2; A1, 0–70 min; A2, 70–140 min), 60 (B1 and B2; B1, 0–70 min; B2, 70–140 min), and 90 (C1 and C2; C1, 0–70 min; C2, 70–140 min). Compared to the control, which was comprised of denatured Parkin (red line), the HPLC chromatograms of the screened SFR extract showed four (K1–K4) peaks that were enhanced due to specific binding with Parkin (black line). The concentrations of Parkin and SFR sample were 1.00 g/L and 10.5 g/L, respectively.

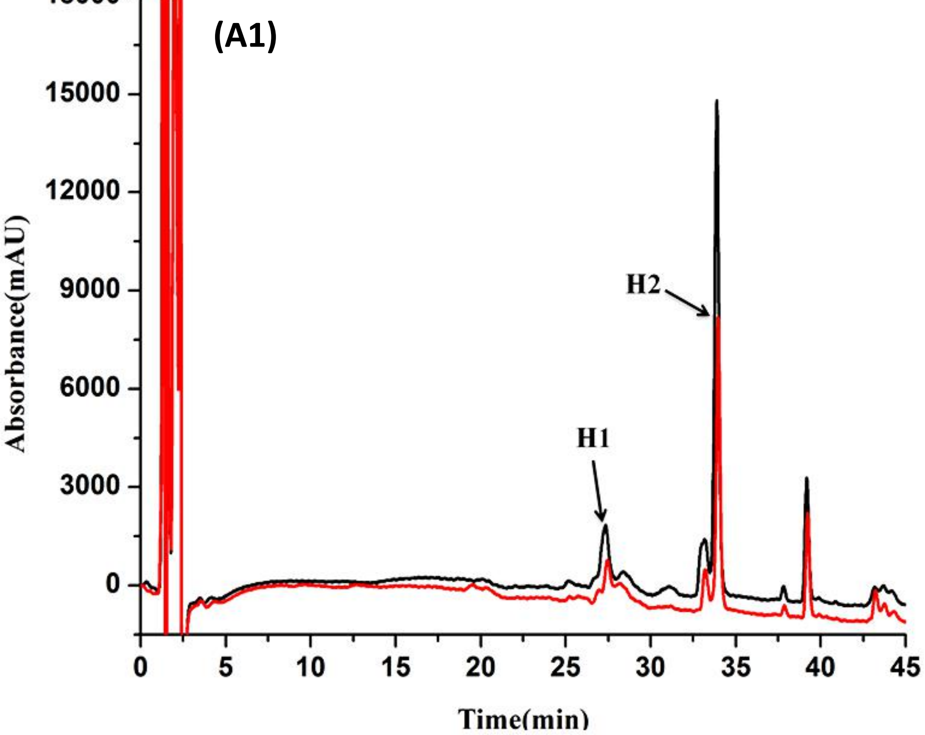
(A1)
H1
H2
Absorbance(mAU)
Time(min)
18000
15000
12000
9000
6000
3000
0
0 5 10 15 20 25 30 35 40 45

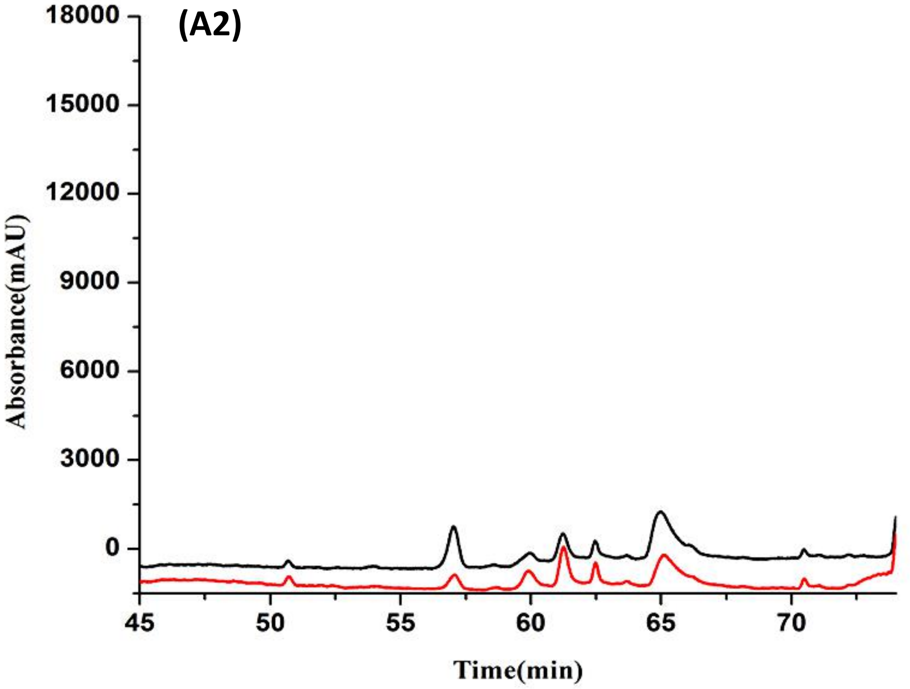
(A2)
Absorbance(mAU)
Time(min)
18000
15000
12000
9000
6000
3000
0
45 50 55 60 65 70

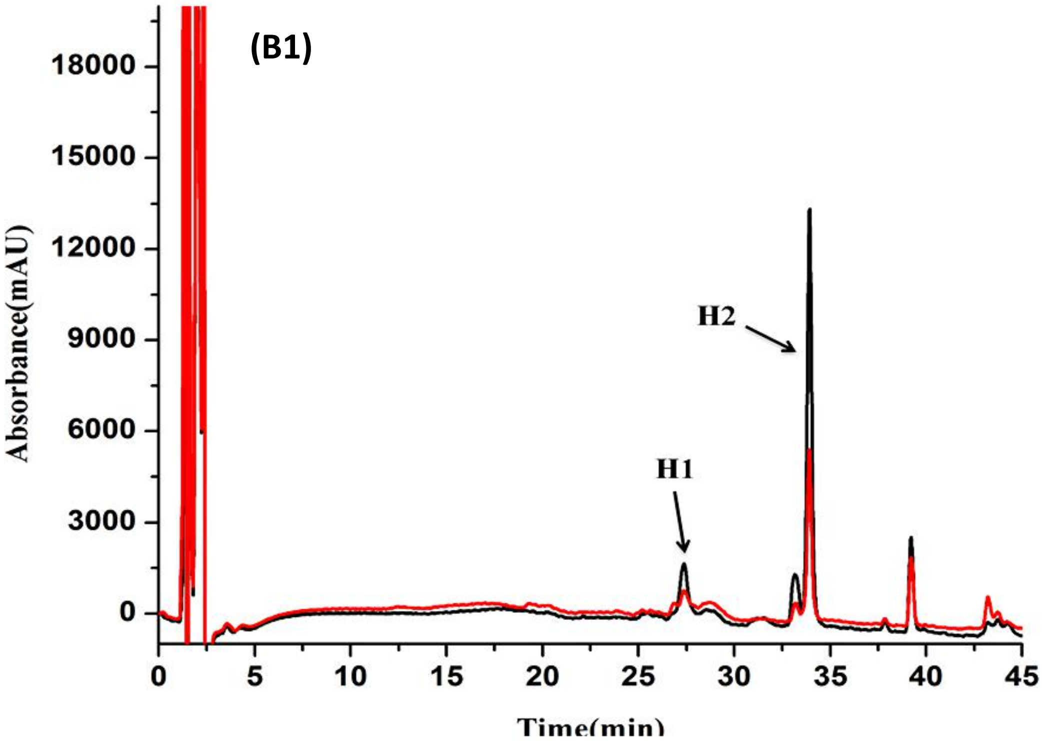
(B1)
H1
H2
Absorbance(mAU)
18000
15000
12000
9000
6000
3000
0
0
5
10
15
20
25
30
35
40
45
Time(min)

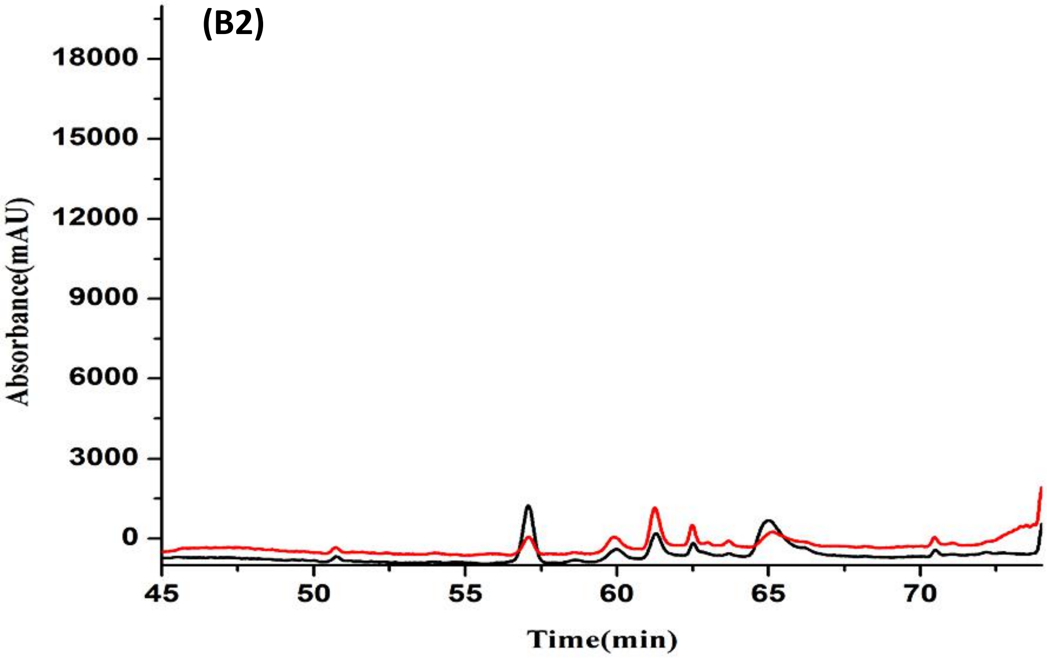
(B2)
Absorbance(mAU)
18000
15000
12000
9000
6000
3000
0
45
50
55
60
65
70
Time(min)

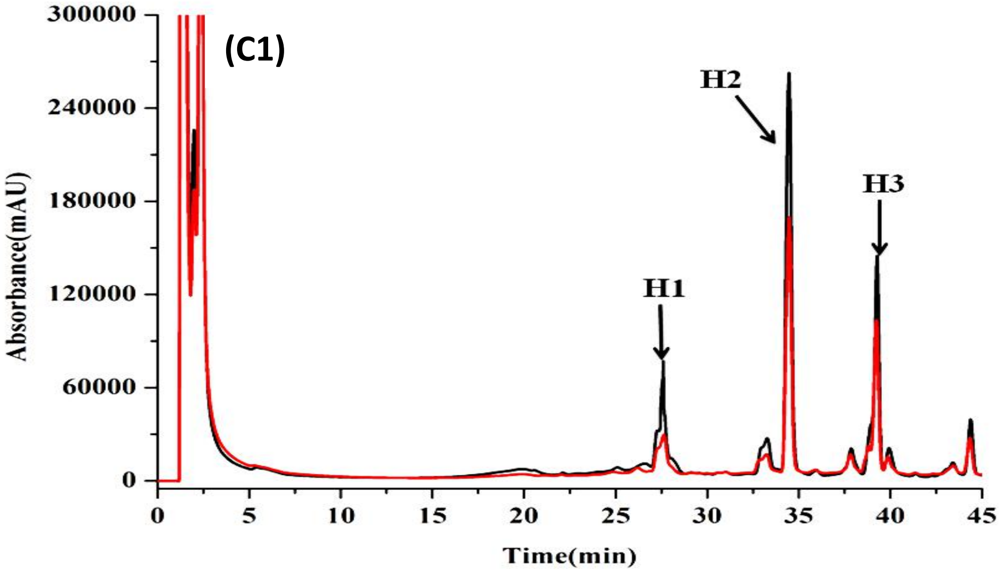

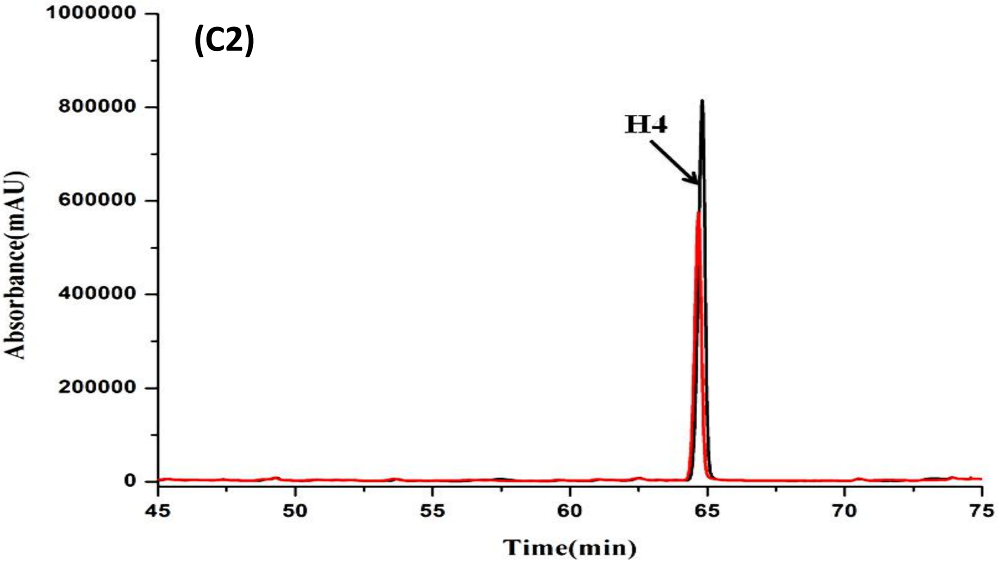

FIGURE S5. Effect of Parkin concentration on fishing of Parkin ligands from PCRR extract. The concentrations of Parkin were 0.25 (A1 and A2; A1, 0–45 min; A2, 45–75 min), 0.50 (B1 and B2; B1, 0–45 min; B2, 45–75 min). Compared to the control and, 1.00 (C1 and C2; C1, 0–45 min; C2, 45–75 min), which was comprised of denatured Parkin (red line), the HPLC chromatograms of the screened PCRR extract showed four (H1–H4) peaks that were enhanced due to specific binding with Parkin (black line). The concentration of PCRR sample and incubation time were 7.5 g/L and 60 min, respectively.

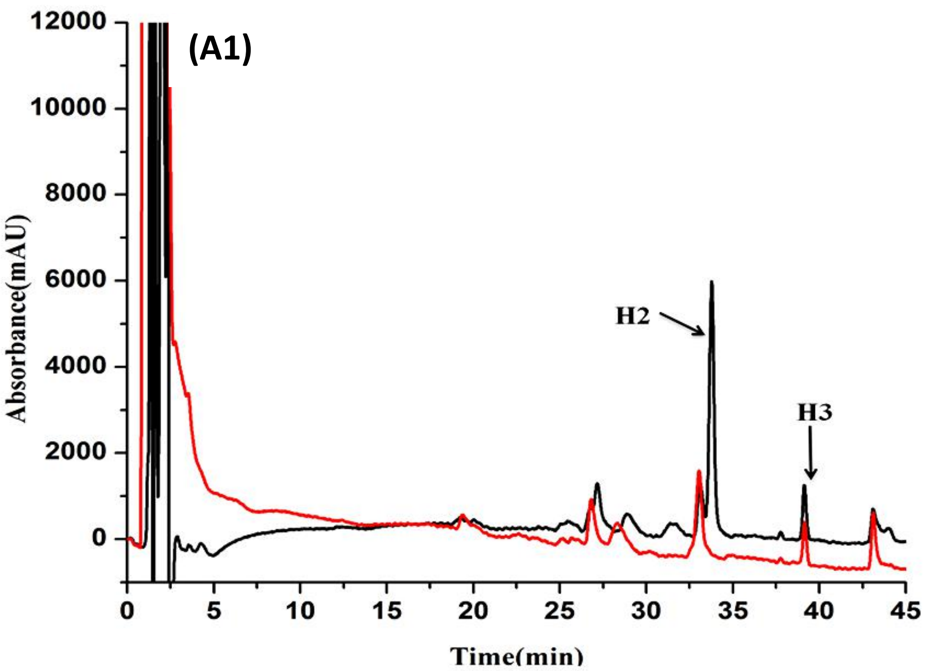
(A1)
Absorbance(mAU)
Time(min)
H2
H3
12000
10000
8000
6000
4000
2000
0
0
5
10
15
20
25
30
35
40
45

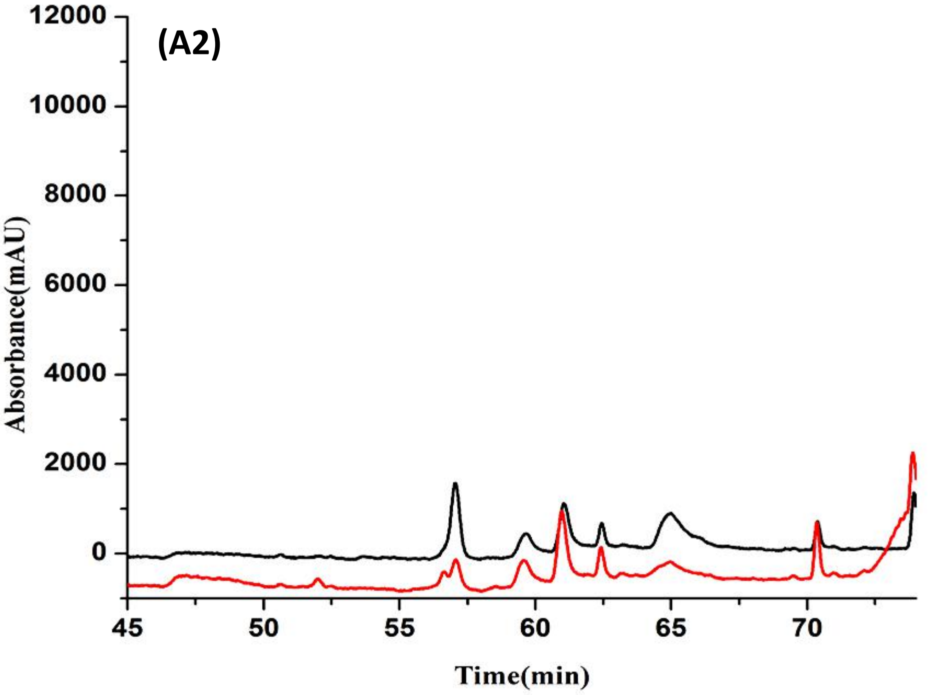
(A2)
Absorbance(mAU)
Time(min)
12000
10000
8000
6000
4000
2000
0
45
50
55
60
65
70

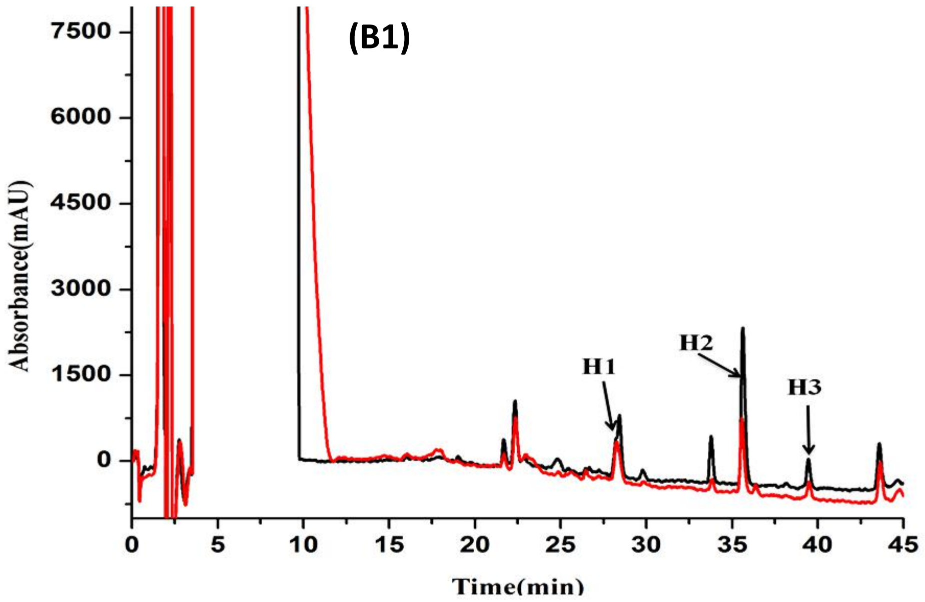
(B1)
Absorbance(mAU)
7500
6000
4500
3000
1500
0
H1
H2
H3
0 5 10 15 20 25 30 35 40 45
Time(min)

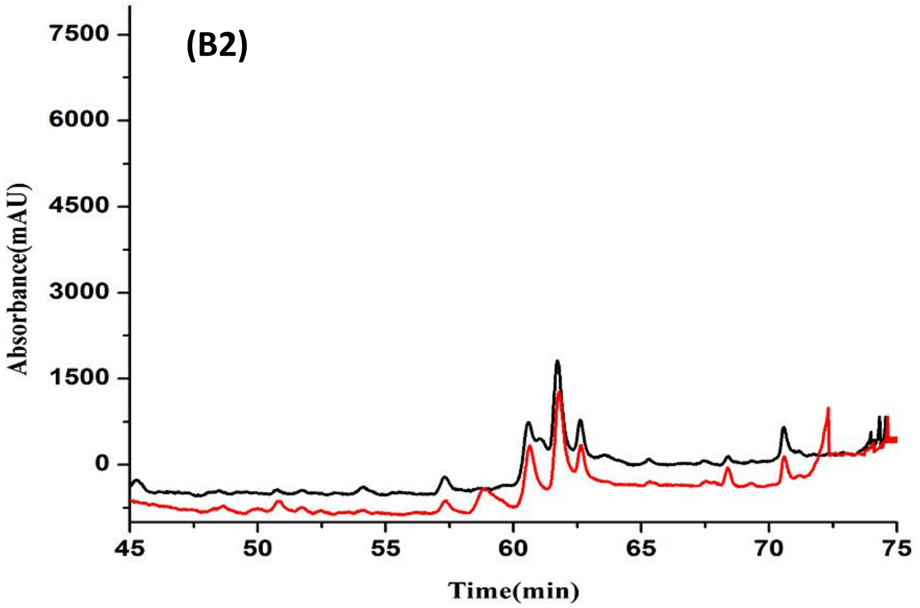
(B2)
Absorbance(mAU)
7500
6000
4500
3000
1500
0
45 50 55 60 65 70 75
Time(min)

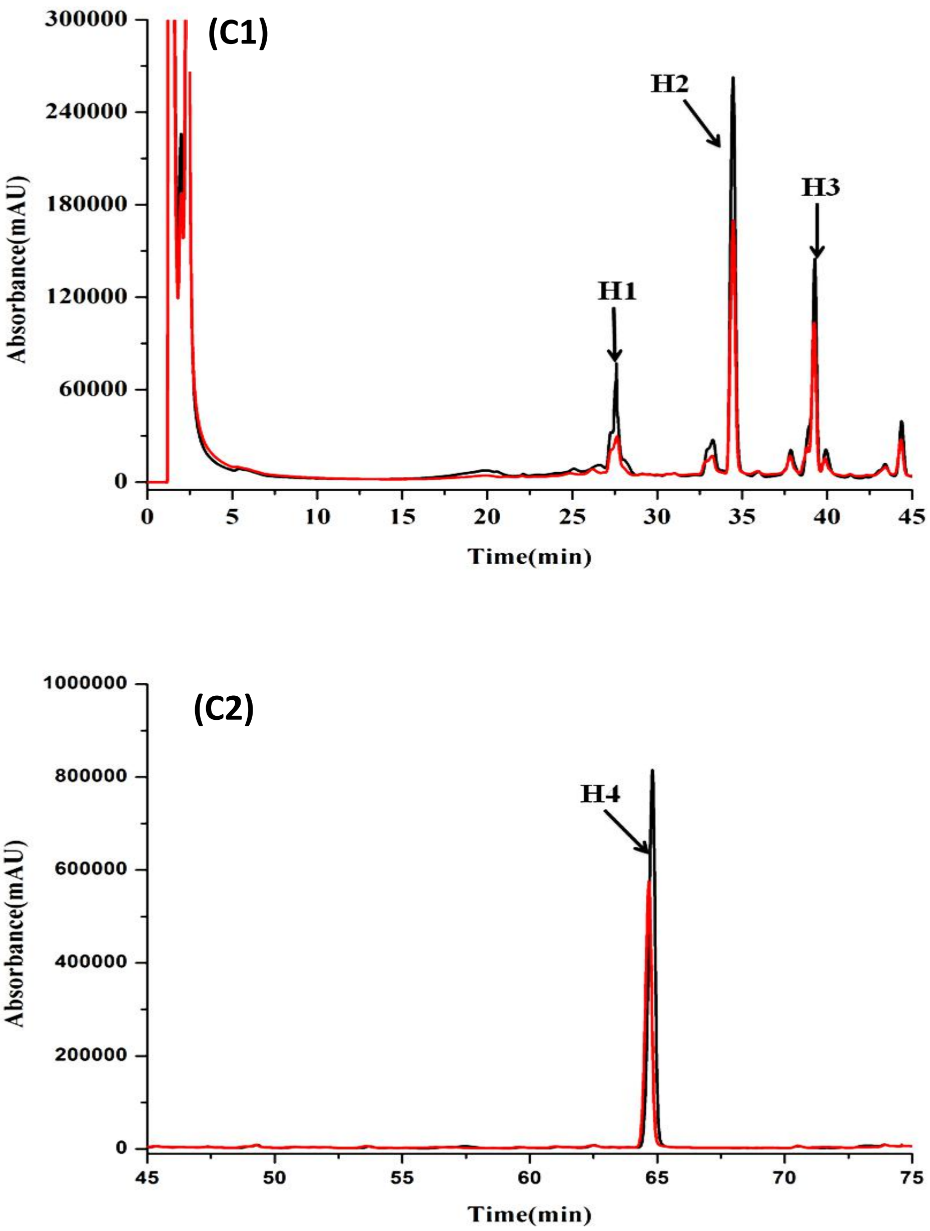

FIGURE S6. Effect of concentration of PCRR sample on fishing of Parkin ligands. The concentrations of PCRR sample were 1.875 (A1 and A2; A1, 0–45 min; A2, 45–75 min), 3.75 (B1 and B2; B1, 0–45 min; B2, 45–75 min) and 7.5 (C1 and C2; C1, 0–45 min; C2, 45–75 min). Compared to the control, which was comprised of denatured Parkin (red line), the HPLC chromatograms of the screened PCRR extract showed four (H1–H4) peaks that were enhanced due to specific binding with Parkin (black line). The concentration of Parkin sample and incubation time were 1.0 g/L and 60 min, respectively.

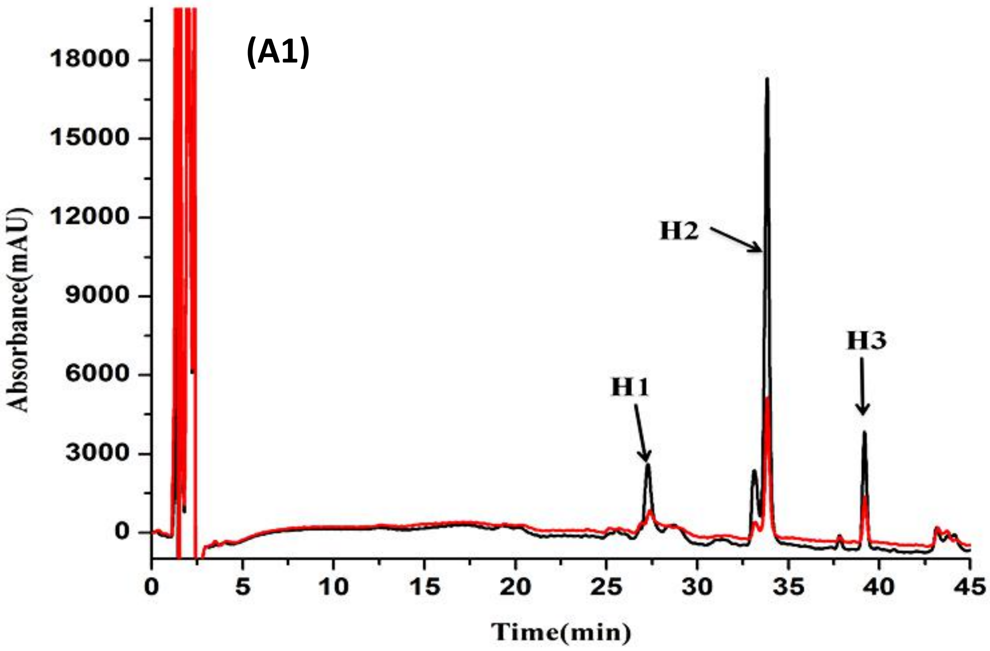

(A1)
H1
H2
H3
Absorbance(mAU)
Time(min)

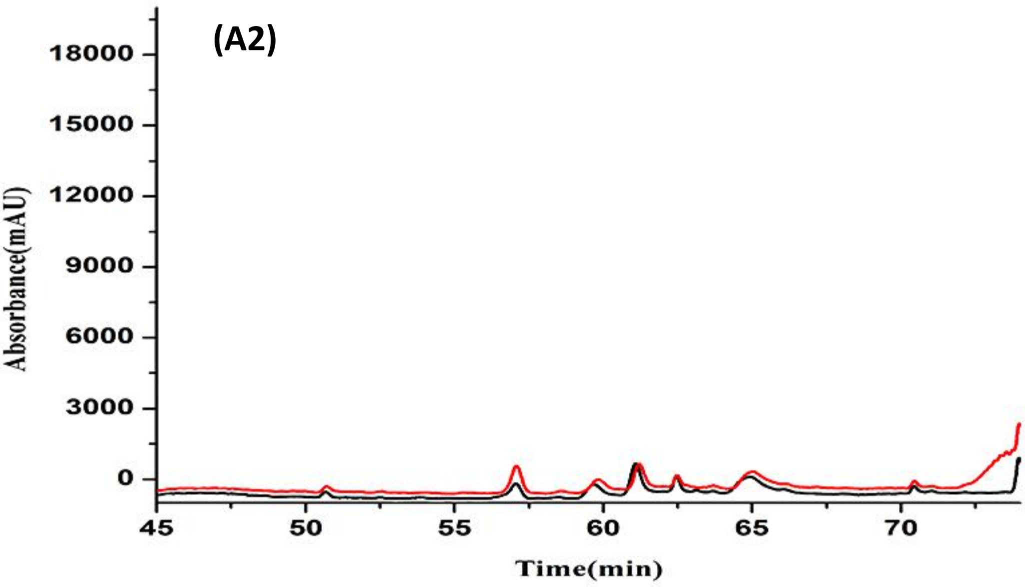

(A2)
Absorbance(mAU)
Time(min)

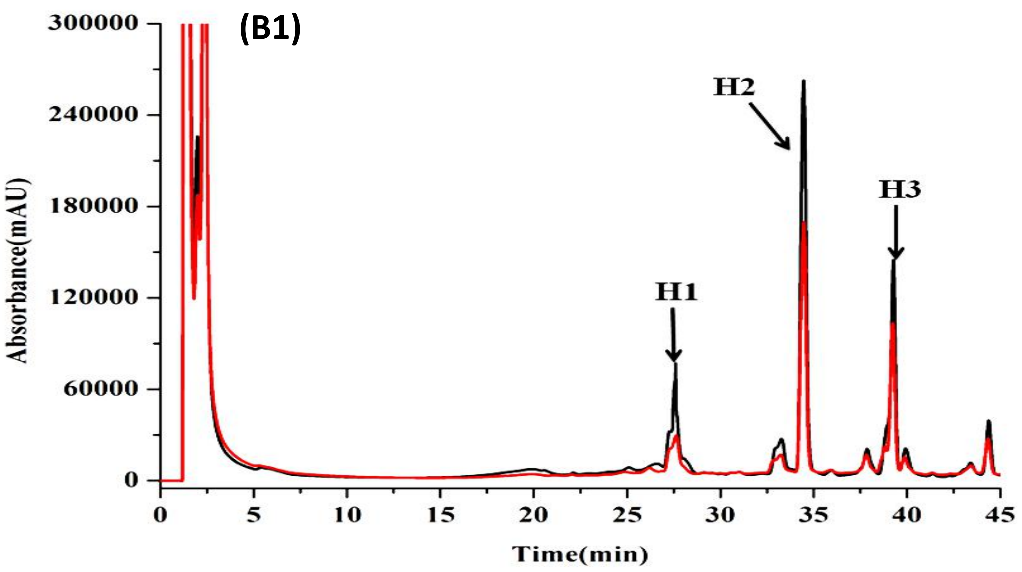
(B1)
H1
H2
H3
Absorbance(mAU)
300000
240000
180000
120000
60000
0
0
5
10
15
20
25
30
35
40
45
Time(min)

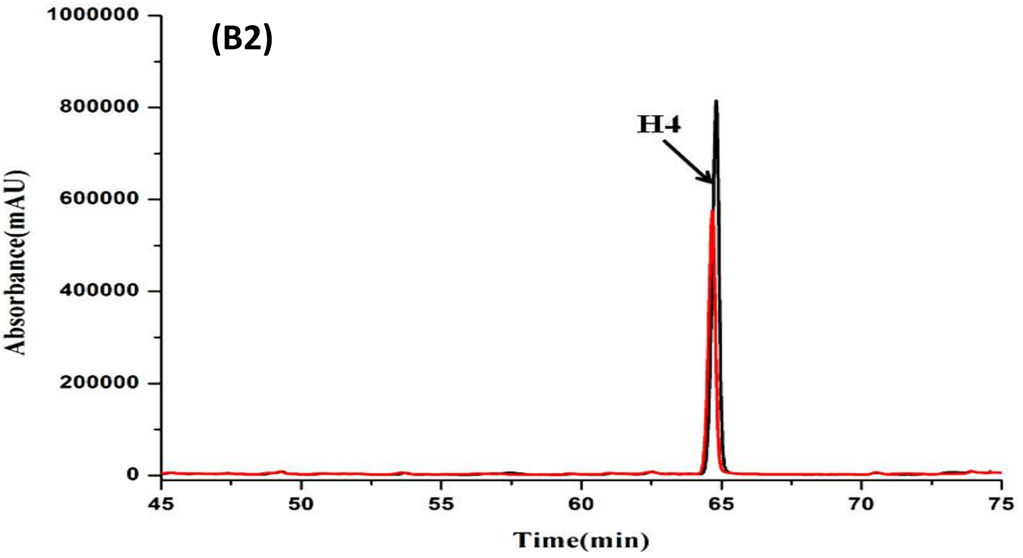
(B2)
H4
Absorbance(mAU)
1000000
800000
600000
400000
200000
0
45
50
55
60
65
70
75
Time(min)

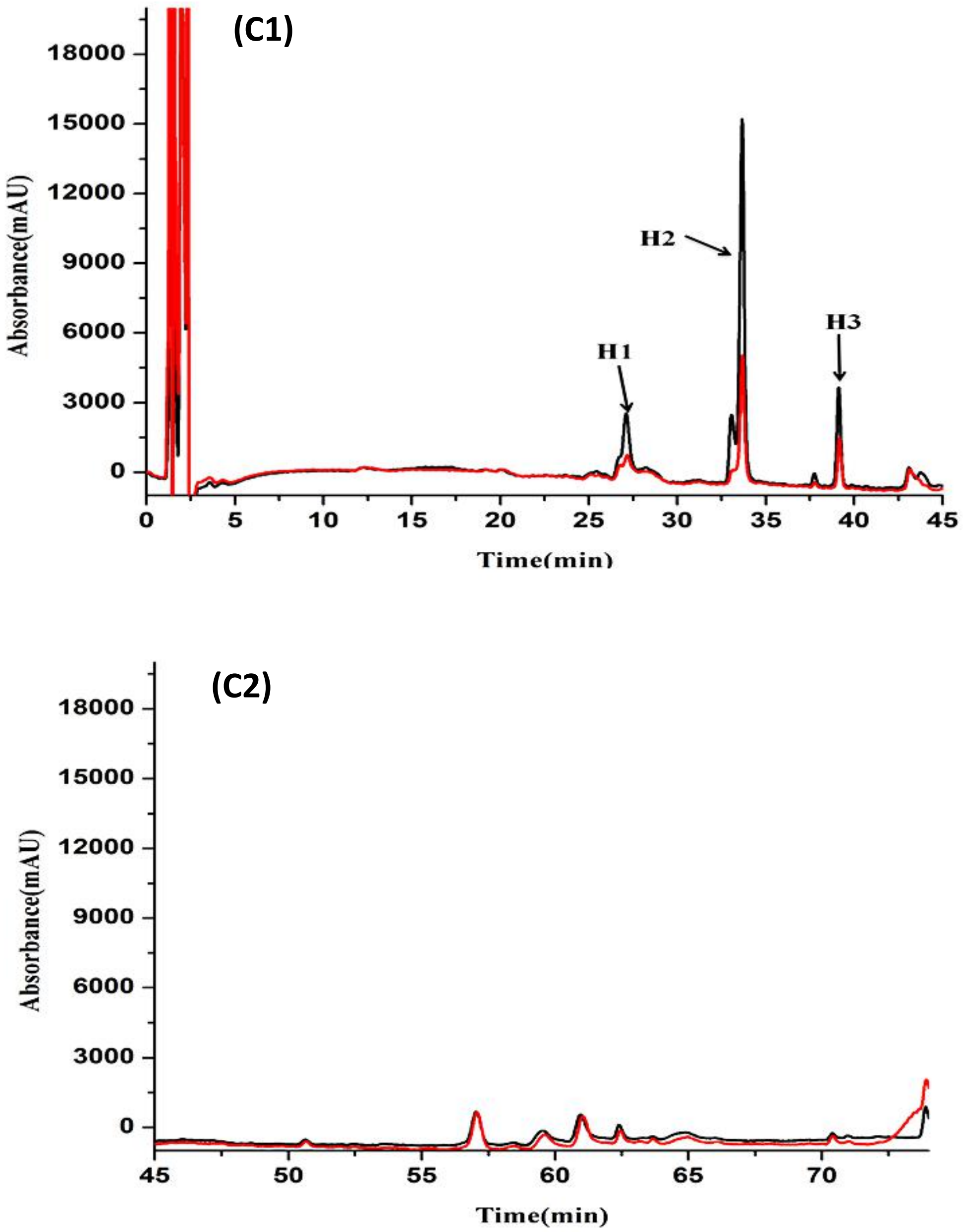

FIGURE S7. Effect of incubation time on fishing of Parkin ligands from PCRR extract. Incubation times were 30 (A1 and A2; A1, 0–45 min; A2, 45–75 min), 60 (B1 and B2; B1, 0–45 min; B2, 45–75 min) and 90 (C1 and C2; C1, 0–45 min; C2, 45–75 min). Compared to the control, which was comprised of denatured Parkin (red line), the HPLC chromatograms of the screened PCRR extract showed four (H1–H4) peaks that were enhanced due to specific binding with Parkin (black line). The concentrations of Parkin and PCRR sample were 1.00 g/L and 7.5 g/L, respectively.

Table S1. HPLC–DAD conditions for all analyzed samples

Sample	Chromatographic column	Mobile phase	Eluent gradient	Injection volume (μL)	Column temperature (°C)	Flow rate (mL/min)	Detection wavelength (nm)
Dithiothreitol	shim-pack XR-ODS (2.0mm×10cm, 2.2 μm)	Water (A) and methanol (B)	0 min, 100%B; 2 min, 95%B; 4 min, 85%B; 7 min, 80%B; 10 min, 70%B; 15 min, 100%B	2	30	0.2	210
DL-selenomethionine	shim-pack XR-ODS (2.0mm×10cm, 2.2 μm)	Water (A) and acetonitrile (B)	0 min, 80%B; 3 min, 60%B; 4 min, 40%B; 5 min, 20%B; 10 min, 80%B	2	30	0.2	210
Amoxicillin	shim-pack XR-ODS (2.0mm×10cm, 2.2 μm)	Water and methanol (85:15 v/v)	Isocratic elution	2	30	0.2	210
Fenofibrate	shim-pack XR-ODS (2.0mm×10cm, 2.2 μm)	Water and acetonitrile (30:70 v/v)	Isocratic elution	2	30	0.2	286
Mixed reference solution (comprised of dithiothreitol, dl-selenomethionine, amoxicillin and fenofibrate)	shim-pack XR-ODS (2.0mm×10cm, 2.2 μm)	Water (A) and acetonitrile (B)	0.01 min, 0.2%B; 2 min, 2%B; 5 min, 8%B; 8 min, 14%B; 11.5 min, 18%B; 14 min, 70%B; 16 min, 80%B; 20 min, 95%B; 22 min, 0.2%B	5	30	0.2	210
SFR extract	shim-pack XR-ODS (2.0mm×10cm, 2.2 μm)	Water (A) and acetonitrile (B)	0 min, 2%B; 15 min, 5%B; 30 min, 15%B; 40 min, 20%B; 60 min, 25%B; 75 min, 35%B; 90 min, 40%B; 105 min, 45%B; 120 min, 55%B; 130 min	5	30	0.2	254

			65B%; 140min 85%B				
PCRR extract	shim-pack XR-ODS (2.0mm×10cm, 2.2 μm)	Water (A) and acetonitrile (B)	0 min, 5%B; 10 min, 15%B; 20 min, 20%B; 35 min, 30%B; 55 min, 40%B; 70 min, 60%B; 75 min, 100%B	5	30	0.2	210

Table S2. LC/MS data for the four reference solutions.

Reference	t_R (min)	P [a] (%, n=3)	UV λmax (nm)	$[M+H]^+$ m/z	ESI-MS^n(+) m/z (abundance)	$[M-H]^+$ m/z	ESI-MS^n(-) m/z (abundance)
DL-Dithiothreitol	2.341	56.3 ± 15.5	204	155.25	—	—	MS^2(153):117(100),151(50),105(42),88(14)
DL-selenomethionine	11.926	37.1 ± 12.2	202	197.11	MS^2(196):181(100),153(45),134(25),108(20)	—	—
Amoxicillin	14.106	12 ± 2.3	270	366.1084	MS^2(366):114(100),208(17)	—	—
Fenofibrate	5.325	6.1 ± 7.4	286	361.1	MS^2(361):232(100),138(10)	—	—

[a]ΔP was calculated using the following equation:

$\Delta P= (Pe\text{-}Pc)/Pe\times 100$, where Pe and Pc are the peak areas in the experiment and control, respectively. Data were obtained from 3 independent experiments and are expressed as the mean± SD.

Table S3. LC/MS data and assignment of four Parkin ligands in SFR extract.

NO.	t_R(min)	Δ*P*[b] (%,n=3)	UV λmax (nm)	$[M+H]^+$ $[M+Na]^+$m/z	ESI-MS^n(+) m/z (abundance)	$[M-H]^+$m/z	ESI-MS^n(-) m/z (abundance)	Predicted formula	Meas (m/z)	Pred (m/z)	Diff (ppm)	DBE	Assigned identification
K1	79.152		289	355.14	MS^2(355):179(100),299(23),235(21),193(2) MS^3(179):109(100)	353.1367	MS^2(353):233(100),218(13),232(11),247(9),234(6) MS^3(233):218(100),217(76),189(76),163(76)	$C_{21}H_{22}O_5$	353.1367	353.1394	-7.65	11	7,4‘-dihydroxy-5-methoxy-8-(γ,γ-dimethylallyl)-flavanone
K2[a]	86.152	27 ± 7.2	198,287	455.1983	MS^2(455):303(100),179(39),304(20),313(4) MS^3(303):179(100),180(7),178(2)	453.1932	MS^2(453):275(100),177(99),421(36),149(30),276(18),178(13),303(11),455(11),422(10),435(10) MS^3(275):151(100),124(62),139(49),191(21),137(11),139(11),140(7)	$C_{26}H_{30}O_7$	453.1932	453.1919	2.87	12	Kushenol I
K3[a]	90.763	37.8 ± 12.4	292	439.1657	MS^2(439):315(100),316(14),440(3) MS^3(315):316(74),241(59),165(49),217(43),163(35),213(34),149(32),187(21),273(20),185(16),199(15)	437.1603	MS^2(437):287(100),261(62),438(50),161(39),313(39),314(35),439(33),325(29),288(24),210(23),241(21),275(15),314(14),299(14),243(13),262(13),176(11),190(11),285(11),437(10),419(10)	$C_{26}H_{30}O_6$	437.1603	437.1606	-0.69	12	Kurarinone
K4[a]	104.494	22.9 ± 13.1	233,291	425.1897	MS^2(425):283(100),301(96),289(5),283(1) MS^3(283):213(100),185(61),255(35),187(34),227(28),256(19),254(3)157(3)	423.1812	MS^2(423):261(100),262(18),161(10),261(7),260(4) MS^3(261):149(100),137(82),192(79)	$C_{25}H_{28}O_6$	423.1812	423.1813	0.24	12	Sophoraflavanone G

[a] Compared with reference solutions.

[b] *P* was calculated using the following equation:

$P = (P_e - P_c)/P_e \times 100$, where P_e and P_c are the peak areas in the experiment and control, respectively. Data were obtained from 3 independent experiments and are expressed as the mean ± SD.

Table S4. LC/MS data and assignment of four Parkin ligands in PCRR extract.

NO.	t_R(min)	ΔP^b (%,n=3)	UV λmax (nm)	$[M+H]^+$ $[M+Na]^+$m/z	ESI-MS^n(+) m/z (abundance)	$[M-H]^+$ m/z	ESI-MS^n(-) m/z (abundance)	Predicted formula	Meas (m/z)	Pred (m/z)	Diff (ppm)	DBE	Assigned identification
H1	27.627		236	409.1458	MS2(409):247(100),229(28),205(1),336(1) MS3(247):229(100),205(12),214(12)	407.1332	MS2(407):245(100),246(18),230(16),244(8),245(6) MS3(245):230(100),215(6),230(5),229(4),231(3)	C20H24O9	407.1332	407.1348	3.93	9	Torachrysone-8-O-glucoside
H2[a]	34.646	65.8 ± 27.1	222,272,424	271.0488	MS2(271):229(100),201(40),173(11),197(8),187(6),230(6) MS3(229):187(100),129(43),135(43),145(43),188(49),201(43)	269.0450	MS2(269):225(100)	C15H10O5	269.0450	269.0455	1.86	0	Apigenin
H3	39.273	28.4 ± 19.4	223,270,425	285.0663	MS2(285):211(100),242(72),253(31),213(27),270(24), 239(23),257(23),151(22),225(17),269(15) MS3(285):168(100)	283.0581	MS2(283):240(100),253(31),241(11),239(6) MS3(240):184(100),212(90),197(62),213(60)	C16H12O5	283.0581	283.0612	-10.95	11	Unidentified
H4[a]	64.811	38.1 ± 31.8	222,266,286,439	271.0475	MS2(271):229(100),201(22),173(17),197(9),187(9),230(9) MS3(229):173(100),187(74),201(66),117(66),145(49)	269.0452	MS2(269):225(100),270(35),241(26),226(20),181(13),197(13), 210(10),271(9),182(7),225(7) MS3(225):210(100),181(70),195(56),182(29),197(21),180(17)	C15H10O5	269.0454	269.0455	-0.37	11	Emodin

[a] Compared with reference solutions.

[b] P was calculated using the following equation:

$P = (P_e - P_c)/P_e \times 100$, where P_e and P_c are the peak areas in the experiment and control, respectively. Data were obtained from 3 independent experiments and are expressed as the mean ± SD.
